# Supplementary material for: Identification and Full Genome Analysis of the First Putative Virus of Sea Buckthorn (Hippophae rhamnoides L.)
Source: Microorganisms. 2022 Sep 28;10(10):1933. doi: 10.3390/microorganisms10101933 (PMC9609781; doi:10.3390/microorganisms10101933)
Supplement: Supplementary file 1 [file microorganisms-10-01933-s001.zip › Supplementary Information.pdf]

Microorganisms

Supplementary Data

Identification and Full Genome Analysis of the First Putative Virus of Sea Buckthorn (*Hippophae rhamnoides* L.)

Ina Balke [1,*], Vilija Zeltina [2], Nikita Zrelovs [3], Ieva Kalnciema [2], Gunta Resevica [2], Rebeka Ludviga [1], Juris Jansons [4], Inga Moročko-Bičevska [5], Dalija Segliņa [6] and Andris Zeltins [2,*]

[1] Plant virus protein research group, Latvian Biomedical Research and Study Centre, Ratsupites street 1, k-1, LV-1067 Riga, Latvia
[2] Plant virology group, Latvian Biomedical Research and Study Centre, Ratsupites street 1, k-1, LV-1067 Riga, Latvia
[3] Bioinformatics core facility, Latvian Biomedical Research and Study Centre, Ratsupites street 1, k-1, LV-1067 Riga, Latvia
[4] Cell Biology and Microscopy Core facility, Latvian Biomedical Research and Study Centre, Ratsupites street 1, k-1, LV-1067 Riga, Latvia
[5] Unit of Plant Pathology and Entomology, Institute of Horticulture, Latvia University of Life Sciences and Technologies, Graudu street 1, LV-3701 Dobele, Latvia
[6] Unit of Processing and Biochemistry, Institute of Horticulture, Latvia University of Life Sciences and Technologies, Graudu street 1, LV-3701 Dobele, Latvia

* Correspondence: inab@biomed.lu.lv (I.B.); anze@biomed.lu.lv (A.Z.)

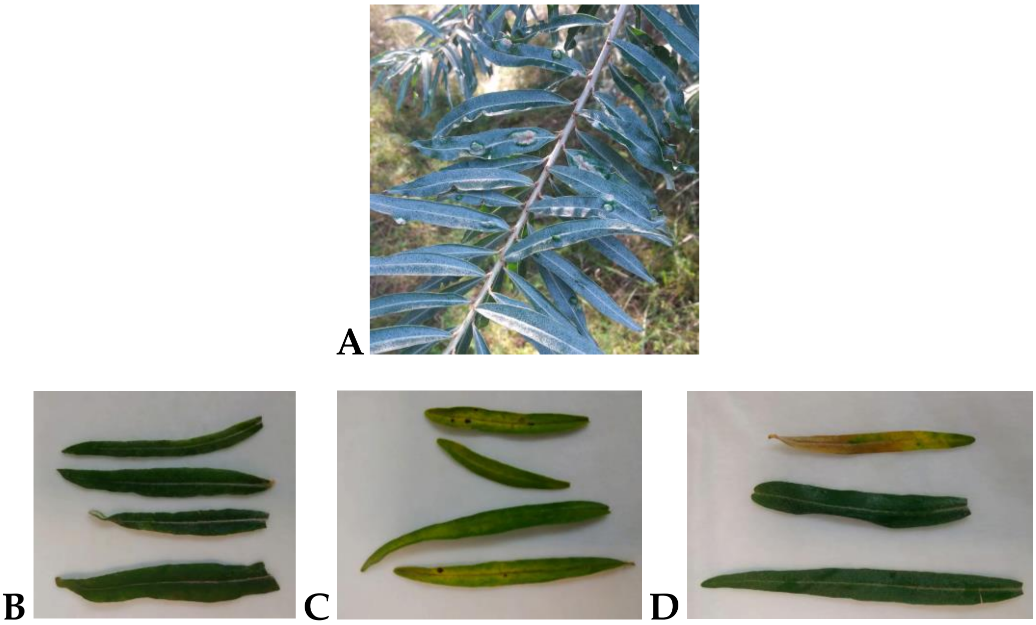

Figure S1. Sea buckthorn leaf samples collected in August 2017 and August 2021. A – representative leaf samples collected in August 2017; B-D – representative leaf samples collected in August 2021.

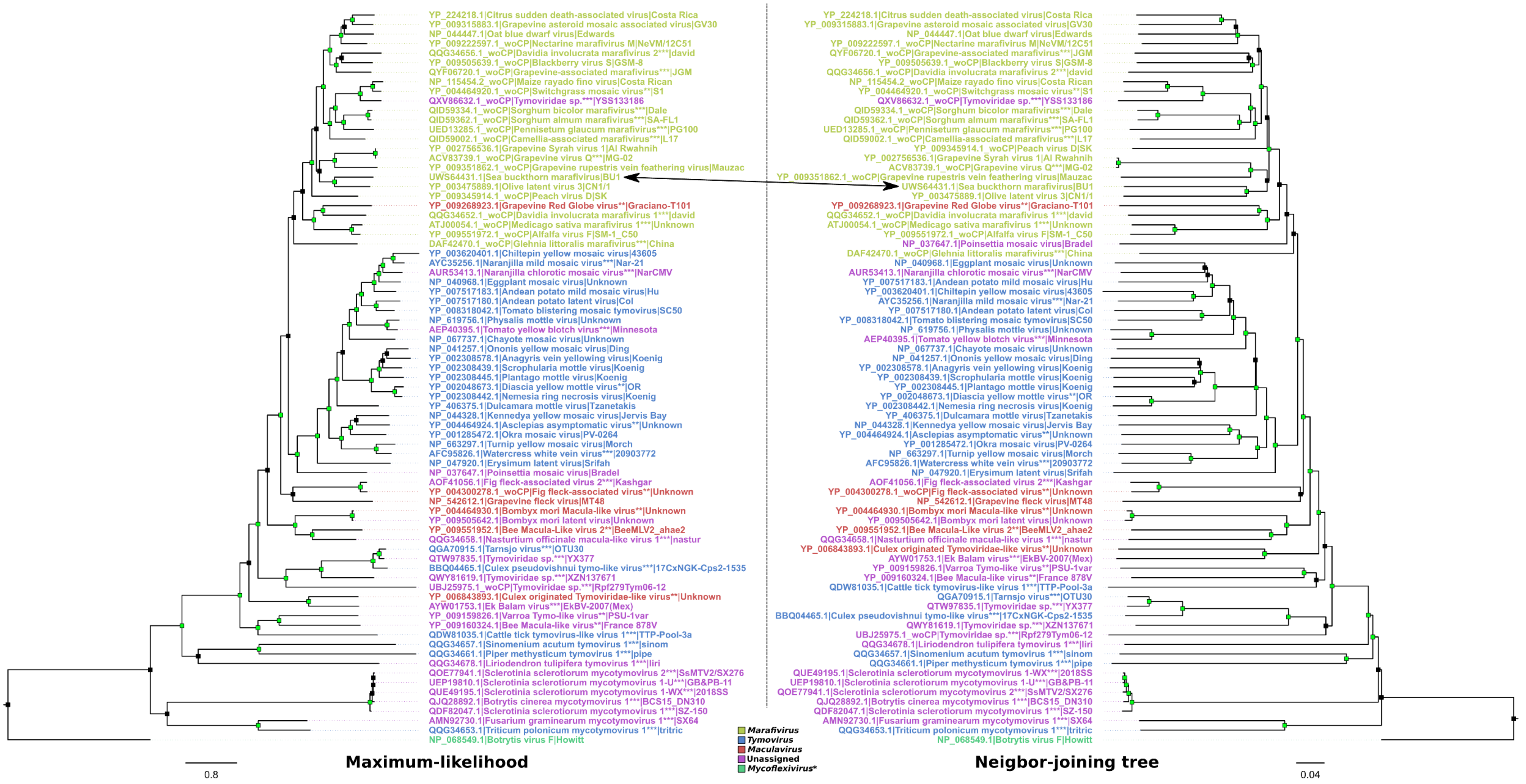
YP_224218.1|Citrus sudden death-associated virus|Costa Rica
YP_009315883.1|Grapevine asteroid mosaic associated virus|GV30
NP_044447.1|Oat blue dwarf virus|Edwards
YP_009222597.1_woCP|Nectarine marafivirus M|NeVM/12C51
QQG34656.1_woCP|Davidia involucrata marafivirus 2***|david
YP_009505639.1_woCP|Blackberry virus S|GSM-8
QYF06720.1_woCP|Grapevine-associated marafivirus***|JGM
NP_115454.2_woCP|Maize rayado fino virus|Costa Rican
YP_004464920.1_woCP|Switchgrass mosaic virus**|S1
QXV86632.1_woCP|Tymoviridae sp.***|YSS133186
QID59334.1_woCP|Sorghum bicolor marafivirus***|Dale
QID59362.1_woCP|Sorghum almum marafivirus***|SA-FL1
UED13285.1_woCP|Pennisetum glaucum marafivirus***|PG100
QID59002.1_woCP|Camellia-associated marafivirus***|L17
YP_002756536.1|Grapevine Syrah virus 1|Al Rwahnih
ACV83739.1_woCP|Grapevine virus Q***|MG-02
YP_009351862.1_woCP|Grapevine rupestris vein feathering virus|Mauzac
UWS64431.1|Sea buckthorn marafivirus|BU1
YP_003475889.1|Olive latent virus 3|CN1/1
YP_009345914.1_woCP|Peach virus D|SK
YP_009268923.1|Grapevine Red Globe virus**|Graciano-T101
QQG34652.1_woCP|Davidia involucrata marafivirus 1***|david
ATJ00054.1_woCP|Medicago sativa marafivirus 1***|Unknown
YP_009551972.1_woCP|Alfalfa virus F|SM-1_C50
DAF42470.1_woCP|Glehnia littoralis marafivirus***|China
YP_003620401.1|Chiltepin yellow mosaic virus|43605
AYC35256.1|Naranjilla mild mosaic virus***|Nar-21
AUR53413.1|Naranjilla chlorotic mosaic virus***|NarCMV
NP_040968.1|Eggplant mosaic virus|Unknown
YP_007517183.1|Andean potato mild mosaic virus|Hu
YP_007517180.1|Andean potato latent virus|Col
YP_008318042.1|Tomato blistering mosaic tymovirus|SC50
NP_619756.1|Physalis mottle virus|Unknown
AEP40395.1|Tomato yellow blotch virus***|Minnesota
NP_067737.1|Chayote mosaic virus|Unknown
NP_041257.1|Ononis yellow mosaic virus|Ding
YP_002308578.1|Anagyris vein yellowing virus|Koenig
YP_002308439.1|Scrophularia mottle virus|Koenig
YP_002308445.1|Plantago mottle virus|Koenig
YP_002048673.1|Diascia yellow mottle virus**|OR
YP_002308442.1|Nemesia ring necrosis virus|Koenig
YP_406375.1|Dulcamara mottle virus|Tzanetakis
NP_044328.1|Kennedya yellow mosaic virus|Jervis Bay
YP_004464924.1|Asclepias asymptomatic virus**|Unknown
YP_001285472.1|Okra mosaic virus|PV-0264
NP_663297.1|Turnip yellow mosaic virus|Morch
AFC95826.1|Watercress white vein virus***|20903772
NP_047920.1|Erysimum latent virus|Srifah
NP_037647.1|Poinsettia mosaic virus|Bradel
AOF41056.1|Fig fleck-associated virus 2***|Kashgar
YP_004300278.1_woCP|Fig fleck-associated virus**|Unknown
NP_542612.1|Grapevine fleck virus|MT48
YP_004464930.1|Bombyx mori Macula-like virus**|Unknown
YP_009505642.1|Bombyx mori latent virus|Unknown
YP_009551952.1|Bee Macula-Like virus 2**|BeeMLV2_ahae2
QQG34658.1|Nasturtium officinale macula-like virus 1***|nastur
QGA70915.1|Tarnsjo virus***|OTU30
QTW97835.1|Tymoviridae sp.***|YX377
BBQ04465.1|Culex pseudovishnui tymo-like virus***|17CxNGK-Cps2-1535
QWY81619.1|Tymoviridae sp.***|XZN137671
UBJ25975.1_woCP|Tymoviridae sp.***|Rpf279Tym06-12
YP_006843893.1|Culex originated Tymoviridae-like virus**|Unknown
AYW01753.1|Ek Balam virus***|EkBV-2007(Mex)
YP_009159826.1|Varroa Tymo-like virus**|PSU-1var
YP_009160324.1|Bee Macula-like virus**|France 878V
QDW81035.1|Cattle tick tymovirus-like virus 1***|TTP-Pool-3a
QQG34657.1|Sinomenium acutum tymovirus 1***|sinom
QQG34661.1|Piper methysticum tymovirus 1***|pipe
QQG34678.1|Liriodendron tulipifera tymovirus 1***|liri
QOE77941.1|Sclerotinia sclerotiorum mycotymovirus 2***|SsMTV2/SX276
UEP19810.1|Sclerotinia sclerotiorum mycotymovirus 1-U***|GB&PB-11
QUE49195.1|Sclerotinia sclerotiorum mycotymovirus 1-WX***|2018SS
QJQ28892.1|Botrytis cinerea mycotymovirus 1***|BCS15_DN310
QDF82047.1|Sclerotinia sclerotiorum mycotymovirus 1***|SZ-150
AMN92730.1|Fusarium graminearum mycotymovirus 1***|SX64
QQG34653.1|Triticum polonicum mycotymovirus 1***|tritric
NP_068549.1|Botrytis virus F|Howitt
0.8
Maximum-likelihood
YP_224218.1|Citrus sudden death-associated virus|Costa Rica
YP_009315883.1|Grapevine asteroid mosaic associated virus|GV30
NP_044447.1|Oat blue dwarf virus|Edwards
YP_009222597.1_woCP|Nectarine marafivirus M|NeVM/12C51
QYF06720.1_woCP|Grapevine-associated marafivirus***|JGM
YP_009505639.1_woCP|Blackberry virus S|GSM-8
QQG34656.1_woCP|Davidia involucrata marafivirus 2***|david
NP_115454.2_woCP|Maize rayado fino virus|Costa Rican
YP_004464920.1_woCP|Switchgrass mosaic virus**|S1
QXV86632.1_woCP|Tymoviridae sp.***|YSS133186
QID59334.1_woCP|Sorghum bicolor marafivirus***|Dale
QID59362.1_woCP|Sorghum almum marafivirus***|SA-FL1
UED13285.1_woCP|Pennisetum glaucum marafivirus***|PG100
QID59002.1_woCP|Camellia-associated marafivirus***|L17
YP_009345914.1_woCP|Peach virus D|SK
YP_002756536.1|Grapevine Syrah virus 1|Al Rwahnih
ACV83739.1_woCP|Grapevine virus Q***|MG-02
YP_009351862.1_woCP|Grapevine rupestris vein feathering virus|Mauzac
UWS64431.1|Sea buckthorn marafivirus|BU1
YP_003475889.1|Olive latent virus 3|CN1/1
YP_009268923.1|Grapevine Red Globe virus**|Graciano-T101
QQG34652.1_woCP|Davidia involucrata marafivirus 1***|david
ATJ00054.1_woCP|Medicago sativa marafivirus 1***|Unknown
YP_009551972.1_woCP|Alfalfa virus F|SM-1_C50
NP_037647.1|Poinsettia mosaic virus|Bradel
DAF42470.1_woCP|Glehnia littoralis marafivirus***|China
NP_040968.1|Eggplant mosaic virus|Unknown
AUR53413.1|Naranjilla chlorotic mosaic virus***|NarCMV
YP_007517183.1|Andean potato mild mosaic virus|Hu
YP_003620401.1|Chiltepin yellow mosaic virus|43605
AYC35256.1|Naranjilla mild mosaic virus***|Nar-21
YP_007517180.1|Andean potato latent virus|Col
YP_008318042.1|Tomato blistering mosaic tymovirus|SC50
NP_619756.1|Physalis mottle virus|Unknown
AEP40395.1|Tomato yellow blotch virus***|Minnesota
NP_067737.1|Chayote mosaic virus|Unknown
NP_041257.1|Ononis yellow mosaic virus|Ding
YP_002308578.1|Anagyris vein yellowing virus|Koenig
YP_002308439.1|Scrophularia mottle virus|Koenig
YP_002308445.1|Plantago mottle virus|Koenig
YP_002048673.1|Diascia yellow mottle virus**|OR
YP_002308442.1|Nemesia ring necrosis virus|Koenig
YP_406375.1|Dulcamara mottle virus|Tzanetakis
NP_044328.1|Kennedya yellow mosaic virus|Jervis Bay
YP_004464924.1|Asclepias asymptomatic virus**|Unknown
YP_001285472.1|Okra mosaic virus|PV-0264
NP_663297.1|Turnip yellow mosaic virus|Morch
AFC95826.1|Watercress white vein virus***|20903772
NP_047920.1|Erysimum latent virus|Srifah
AOF41056.1|Fig fleck-associated virus 2***|Kashgar
YP_004300278.1_woCP|Fig fleck-associated virus**|Unknown
NP_542612.1|Grapevine fleck virus|MT48
YP_004464930.1|Bombyx mori Macula-like virus**|Unknown
YP_009505642.1|Bombyx mori latent virus|Unknown
YP_009551952.1|Bee Macula-Like virus 2**|BeeMLV2_ahae2
QQG34658.1|Nasturtium officinale macula-like virus 1***|nastur
YP_006843893.1|Culex originated Tymoviridae-like virus**|Unknown
AYW01753.1|Ek Balam virus***|EkBV-2007(Mex)
YP_009159826.1|Varroa Tymo-like virus**|PSU-1var
YP_009160324.1|Bee Macula-like virus**|France 878V
QDW81035.1|Cattle tick tymovirus-like virus 1***|TTP-Pool-3a
QGA70915.1|Tarnsjo virus***|OTU30
QTW97835.1|Tymoviridae sp.***|YX377
BBQ04465.1|Culex pseudovishnui tymo-like virus***|17CxNGK-Cps2-1535
QWY81619.1|Tymoviridae sp.***|XZN137671
UBJ25975.1_woCP|Tymoviridae sp.***|Rpf279Tym06-12
QQG34678.1|Liriodendron tulipifera tymovirus 1***|liri
QQG34657.1|Sinomenium acutum tymovirus 1***|sinom
QQG34661.1|Piper methysticum tymovirus 1***|pipe
QUE49195.1|Sclerotinia sclerotiorum mycotymovirus 1-WX***|2018SS
UEP19810.1|Sclerotinia sclerotiorum mycotymovirus 1-U***|GB&PB-11
QOE77941.1|Sclerotinia sclerotiorum mycotymovirus 2***|SsMTV2/SX276
QJQ28892.1|Botrytis cinerea mycotymovirus 1***|BCS15_DN310
QDF82047.1|Sclerotinia sclerotiorum mycotymovirus 1***|SZ-150
AMN92730.1|Fusarium graminearum mycotymovirus 1***|SX64
QQG34653.1|Triticum polonicum mycotymovirus 1***|tritric
NP_068549.1|Botrytis virus F|Howitt
Marafivirus
Tymovirus
Maculavirus
Unassigned
Mycoflexivirus*
Neigbor-joining tree
0.04

Figure S2. Maximum-likelihood and neighbor-joining trees generated using *Tymoviridae* capsid protein sequence. Trees were drawn to scale with branch lengths in units of amino acid substitutions per site. In the case of capsid proteins that were encoded as part of the polyprotein, the last 300 aa of the polyprotein sequence containing presumed CP were used (indicated as "_300last" next to the accession number of a respective sequence). Tree tip labels are in the form of sequence accession number|virus name|strain and are colored based on the genera to which the virus belongs according to the legend. Two asterisks (**) after the virus name indicate that the virus originated from RefSeq and did not yet have a standing in the official virus taxonomy but was included in the analysis based on taxonomy associated with a RefSeq entry. Three asterisks (***) after the virus name indicate that the sequence originated from the Nuccore database and did not yet have a standing in the official virus taxonomy but was included in the analysis based on taxonomy associated with a complete or near-complete genome GenBank entry that was longer than 6000 bases in length. Botrytis virus F, which is a member of the *Gammaflexiviridae* family, genus *Mycoflexivirus*, serves as an outgroup at which the trees are rooted. The black arrow connects the sea buckthorn marafivirus (SBuMV) leaves in both trees.

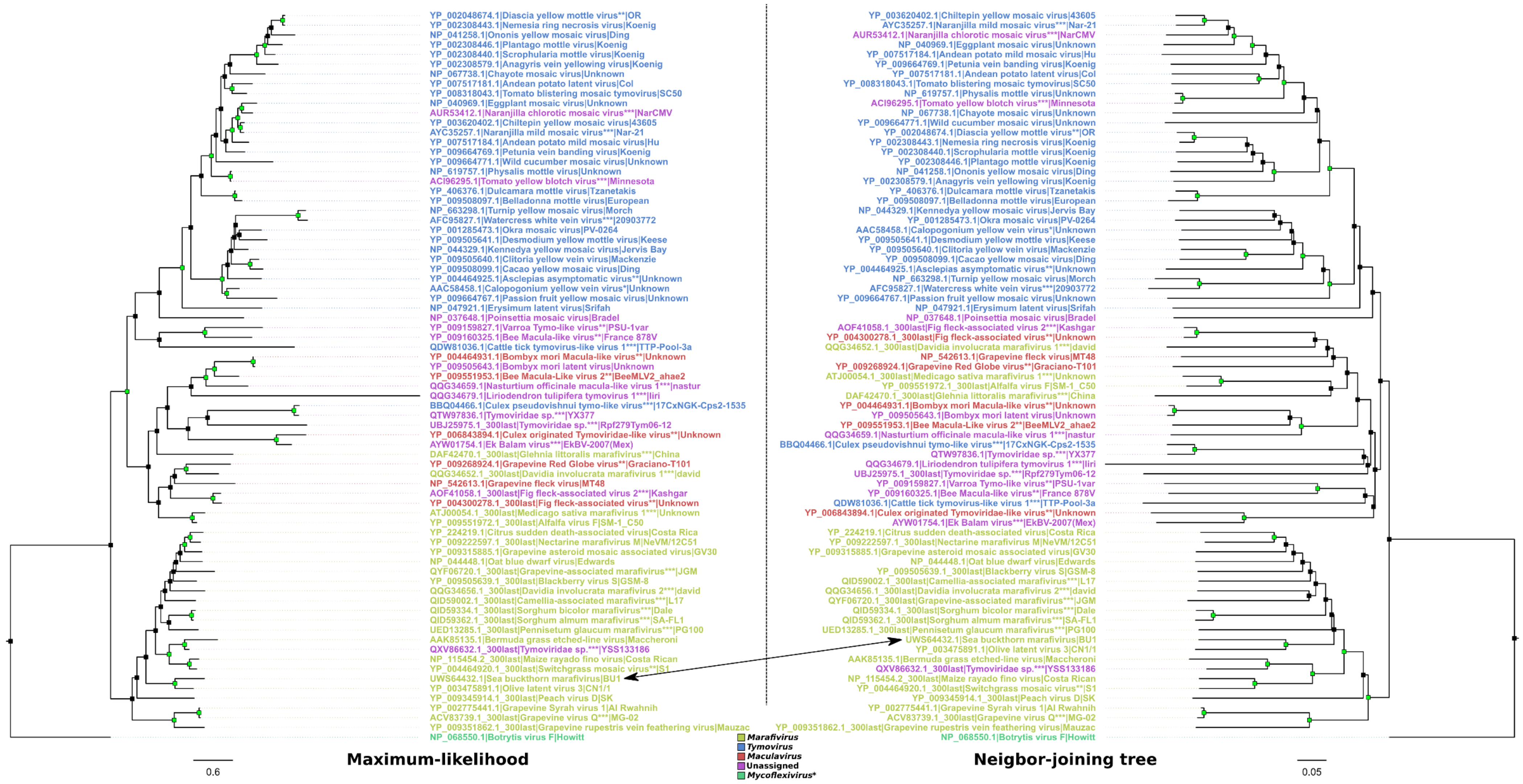

YP_002048674.1|Diascia yellow mottle virus**|OR
YP_002308443.1|Nemesia ring necrosis virus|Koenig
NP_041258.1|Ononis yellow mosaic virus|Ding
YP_002308446.1|Plantago mottle virus|Koenig
YP_002308440.1|Scrophularia mottle virus|Koenig
YP_002308579.1|Anagyris vein yellowing virus|Koenig
NP_067738.1|Chayote mosaic virus|Unknown
YP_007517181.1|Andean potato latent virus|Col
YP_008318043.1|Tomato blistering mosaic tymovirus|SC50
NP_040969.1|Eggplant mosaic virus|Unknown
AUR53412.1|Naranjilla chlorotic mosaic virus***|NarCMV
YP_003620402.1|Chiltepin yellow mosaic virus|43605
AYC35257.1|Naranjilla mild mosaic virus***|Nar-21
YP_007517184.1|Andean potato mild mosaic virus|Hu
YP_009664769.1|Petunia vein banding virus|Koenig
YP_009664771.1|Wild cucumber mosaic virus|Unknown
NP_619757.1|Physalis mottle virus|Unknown
ACI96295.1|Tomato yellow blotch virus***|Minnesota
YP_406376.1|Dulcamara mottle virus|Tzanetakis
YP_009508097.1|Belladonna mottle virus|European
NP_663298.1|Turnip yellow mosaic virus|Morch
AFC95827.1|Watercress white vein virus***|20903772
YP_001285473.1|Okra mosaic virus|PV-0264
YP_009505641.1|Desmodium yellow mottle virus|Keese
NP_044329.1|Kennedya yellow mosaic virus|Jervis Bay
YP_009505640.1|Clitoria yellow vein virus|Mackenzie
YP_009508099.1|Cacao yellow mosaic virus|Ding
YP_004464925.1|Asclepias asymptomatic virus**|Unknown
AAC58458.1|Calopogonium yellow vein virus*|Unknown
YP_009664767.1|Passion fruit yellow mosaic virus|Unknown
NP_047921.1|Erysimum latent virus|Srifah
NP_037648.1|Poinsettia mosaic virus|Bradel
YP_009159827.1|Varroa Tymo-like virus**|PSU-1var
YP_009160325.1|Bee Macula-like virus**|France 878V
QDW81036.1|Cattle tick tymovirus-like virus 1***|TTP-Pool-3a
YP_004464931.1|Bombyx mori Macula-like virus**|Unknown
YP_009505643.1|Bombyx mori latent virus|Unknown
YP_009551953.1|Bee Macula-Like virus 2**|BeeMLV2_ahae2
QQG34659.1|Nasturtium officinale macula-like virus 1***|nastur
QQG34679.1|Liriodendron tulipifera tymovirus 1***|liri
BBQ04466.1|Culex pseudovishnui tymo-like virus***|17CxNGK-Cps2-1535
QTW97836.1|Tymoviridae sp.***|YX377
UBJ25975.1_300last|Tymoviridae sp.***|Rpf279Tym06-12
YP_006843894.1|Culex originated Tymoviridae-like virus**|Unknown
AYW01754.1|Ek Balam virus***|EkBV-2007(Mex)
DAF42470.1_300last|Glehnia littoralis marafivirus***|China
YP_009268924.1|Grapevine Red Globe virus**|Graciano-T101
QQG34652.1_300last|Davidia involucrata marafivirus 1***|david
NP_542613.1|Grapevine fleck virus|MT48
AOF41058.1_300last|Fig fleck-associated virus 2***|Kashgar
YP_004300278.1_300last|Fig fleck-associated virus**|Unknown
ATJ00054.1_300last|Medicago sativa marafivirus 1***|Unknown
YP_009551972.1_300last|Alfalfa virus F|SM-1_C50
YP_224219.1|Citrus sudden death-associated virus|Costa Rica
YP_009222597.1_300last|Nectarine marafivirus M|NeVM/12C51
YP_009315885.1|Grapevine asteroid mosaic associated virus|GV30
NP_044448.1|Oat blue dwarf virus|Edwards
QYF06720.1_300last|Grapevine-associated marafivirus***|JGM
YP_009505639.1_300last|Blackberry virus S|GSM-8
QQG34656.1_300last|Davidia involucrata marafivirus 2***|david
QID59002.1_300last|Camellia-associated marafivirus***|L17
QID59334.1_300last|Sorghum bicolor marafivirus***|Dale
QID59362.1_300last|Sorghum almum marafivirus***|SA-FL1
UED13285.1_300last|Pennisetum glaucum marafivirus***|PG100
AAK85135.1|Bermuda grass etched-line virus|Maccheroni
QXV86632.1_300last|Tymoviridae sp.***|YSS133186
NP_115454.2_300last|Maize rayado fino virus|Costa Rican
YP_004464920.1_300last|Switchgrass mosaic virus**|S1
UWS64432.1|Sea buckthorn marafivirus|BU1
YP_003475891.1|Olive latent virus 3|CN1/1
YP_009345914.1_300last|Peach virus D|SK
YP_002775441.1|Grapevine Syrah virus 1|Al Rwahnih
ACV83739.1_300last|Grapevine virus Q***|MG-02
YP_009351862.1_300last|Grapevine rupestris vein feathering virus|Mauzac
NP_068550.1|Botrytis virus F|Howitt
0.6
Maximum-likelihood
YP_003620402.1|Chiltepin yellow mosaic virus|43605
AYC35257.1|Naranjilla mild mosaic virus***|Nar-21
AUR53412.1|Naranjilla chlorotic mosaic virus***|NarCMV
NP_040969.1|Eggplant mosaic virus|Unknown
YP_007517184.1|Andean potato mild mosaic virus|Hu
YP_009664769.1|Petunia vein banding virus|Koenig
YP_007517181.1|Andean potato latent virus|Col
YP_008318043.1|Tomato blistering mosaic tymovirus|SC50
NP_619757.1|Physalis mottle virus|Unknown
ACI96295.1|Tomato yellow blotch virus***|Minnesota
NP_067738.1|Chayote mosaic virus|Unknown
YP_009664771.1|Wild cucumber mosaic virus|Unknown
YP_002048674.1|Diascia yellow mottle virus**|OR
YP_002308443.1|Nemesia ring necrosis virus|Koenig
YP_002308440.1|Scrophularia mottle virus|Koenig
YP_002308446.1|Plantago mottle virus|Koenig
NP_041258.1|Ononis yellow mosaic virus|Ding
YP_002308579.1|Anagyris vein yellowing virus|Koenig
YP_406376.1|Dulcamara mottle virus|Tzanetakis
YP_009508097.1|Belladonna mottle virus|European
NP_044329.1|Kennedya yellow mosaic virus|Jervis Bay
YP_001285473.1|Okra mosaic virus|PV-0264
AAC58458.1|Calopogonium yellow vein virus*|Unknown
YP_009505641.1|Desmodium yellow mottle virus|Keese
YP_009505640.1|Clitoria yellow vein virus|Mackenzie
YP_009508099.1|Cacao yellow mosaic virus|Ding
YP_004464925.1|Asclepias asymptomatic virus**|Unknown
NP_663298.1|Turnip yellow mosaic virus|Morch
AFC95827.1|Watercress white vein virus***|20903772
YP_009664767.1|Passion fruit yellow mosaic virus|Unknown
NP_047921.1|Erysimum latent virus|Srifah
NP_037648.1|Poinsettia mosaic virus|Bradel
AOF41058.1_300last|Fig fleck-associated virus 2***|Kashgar
YP_004300278.1_300last|Fig fleck-associated virus**|Unknown
QQG34652.1_300last|Davidia involucrata marafivirus 1***|david
NP_542613.1|Grapevine fleck virus|MT48
YP_009268924.1|Grapevine Red Globe virus**|Graciano-T101
ATJ00054.1_300last|Medicago sativa marafivirus 1***|Unknown
YP_009551972.1_300last|Alfalfa virus F|SM-1_C50
DAF42470.1_300last|Glehnia littoralis marafivirus***|China
YP_004464931.1|Bombyx mori Macula-like virus**|Unknown
YP_009505643.1|Bombyx mori latent virus|Unknown
YP_009551953.1|Bee Macula-Like virus 2**|BeeMLV2_ahae2
QQG34659.1|Nasturtium officinale macula-like virus 1***|nastur
BBQ04466.1|Culex pseudovishnui tymo-like virus***|17CxNGK-Cps2-1535
QTW97836.1|Tymoviridae sp.***|YX377
QQG34679.1|Liriodendron tulipifera tymovirus 1***|liri
UBJ25975.1_300last|Tymoviridae sp.***|Rpf279Tym06-12
YP_009159827.1|Varroa Tymo-like virus**|PSU-1var
YP_009160325.1|Bee Macula-like virus**|France 878V
QDW81036.1|Cattle tick tymovirus-like virus 1***|TTP-Pool-3a
YP_006843894.1|Culex originated Tymoviridae-like virus**|Unknown
AYW01754.1|Ek Balam virus***|EkBV-2007(Mex)
YP_224219.1|Citrus sudden death-associated virus|Costa Rica
YP_009222597.1_300last|Nectarine marafivirus M|NeVM/12C51
YP_009315885.1|Grapevine asteroid mosaic associated virus|GV30
NP_044448.1|Oat blue dwarf virus|Edwards
YP_009505639.1_300last|Blackberry virus S|GSM-8
QID59002.1_300last|Camellia-associated marafivirus***|L17
QQG34656.1_300last|Davidia involucrata marafivirus 2***|david
QYF06720.1_300last|Grapevine-associated marafivirus***|JGM
QID59334.1_300last|Sorghum bicolor marafivirus***|Dale
QID59362.1_300last|Sorghum almum marafivirus***|SA-FL1
UED13285.1_300last|Pennisetum glaucum marafivirus***|PG100
UWS64432.1|Sea buckthorn marafivirus|BU1
YP_003475891.1|Olive latent virus 3|CN1/1
AAK85135.1|Bermuda grass etched-line virus|Maccheroni
QXV86632.1_300last|Tymoviridae sp.***|YSS133186
NP_115454.2_300last|Maize rayado fino virus|Costa Rican
YP_004464920.1_300last|Switchgrass mosaic virus**|S1
YP_009345914.1_300last|Peach virus D|SK
YP_002775441.1|Grapevine Syrah virus 1|Al Rwahnih
ACV83739.1_300last|Grapevine virus Q***|MG-02
YP_009351862.1_300last|Grapevine rupestris vein feathering virus|Mauzac
NP_068550.1|Botrytis virus F|Howitt
0.05
Neigbor-joining tree
Marafivirus
Tymovirus
Maculavirus
Unassigned
Mycoflexivirus*

Figure S3. *Tymoviridae* polyprotein aa sequence with maximum-likelihood and neighbor-joining trees. Trees were drawn to scale with branch lengths in units of amino acid substitutions per site. In the case of polyproteins that encoded capsid proteins as well, the last 300 aa of the sequence were truncated (indicated as "_woCP" next to the accession number of a respective sequence). Tree tip labels are in the form of sequence accession number|virus name|strain and are colored based on the genera to which the virus belongs according to the legend. Two asterisks (**) after the virus name indicate that the virus originated from RefSeq and did not yet have a standing in the official virus taxonomy but was included in the analysis on the basis of taxonomy associated with a RefSeq entry. Three asterisks (***) after the virus name indicate that the sequence originated from the Nuccore database and does not yet have a standing in the official virus taxonomy but was included in the analysis on the basis of taxonomy associated with a complete or near-complete genome GenBank entry that was longer than 6000 bases in length. Botrytis virus F, which is a member of the *Gammaflexiviridae* family, genus *Mycoflexivirus*, serves as an outgroup at which the trees are rooted. The black arrow connects the Sea buckthorn marafivirus (SBuMV) leaves in both trees.

File S1. Alignment of marafivirus RP and CP with coded protein domain motifs using PROMALS3D.

```
Conservation:                 9
YP_009505639.1_BlVS      1   MER-----------------SNSNLFASTSACFSLAIPISPLDFSPNAEAESLLP---------------   38
MZ422607.1_GaMV              ----------------------------------------------------------------------
ADD13602.1_OBDV          1   MT-------------------TYAFHPLLPTPTSFAT---------------------------------   18
AYC35261.1_CSDaV         1   MD------------------RISARIPVAPATAGPTVYVPYPHTHPLLPRGVFTSGPIQPRLHFLPHHAQ   52
QLM05437.1_GAMaV         1   MD-------------------ASTPLPSFFHPGSPAVEVAPLPPSPECPLTPGFSLSSPSPSPLSPPPS-   50
YP_009222597.1_NeVM      1   MT-------------------PSNFPHLLARSSPKSSRTPAPTPAPR-----------------------   28
MZ305310.1_PGMV          1   ME--------------------------------------------------------------------    2
QID59334.1_SbMV          1   MA---------------------------------STSDQGPTGTPIWRRMP------------------   19
QID59002.1_CaMaV         1   MEC-----------------SLFTSIIFSWTTSQNNITYSSTPATPVARAEGPYFDPEFAKPHLPGATG-   52
QQG34656.1_DiMV2         1   MS------------------SPSTSSCDFVVSTSLIQDLRAQFLGSGNIKYAPLSASRPTFHDLHPHPF-   51
YP_004464920.1_SwMV      1   MS--------------------------------------------------------------------    2
NP_115454.2_MRFV         1   MS--------------------------------------------------------------------    2
QCC30252.1_PeDV          1   M---------------------------------------------------------------------    1
SBuMV-BU1                1   MA--------------------------ALNPSFASFISTFPSPAPEEYHFTCPSFPLLEDVETSIPIL-   43
YP_003475889.1_OLV-3     1   MAA-----------------SWTSFSSLLSNPLSLAQRILTPPHNPSIPSLPKSGFRSVTSLSDPSDAL-   52
YP_009551972.1_AVF       1   MS-------------------TNFQIIPLSFDDSSPSPSSPPNTPAPSPKIKYITPHLSVRFHLPVKRF-   50
ATJ00054.1_MsMV1         1   MS-------------------TSYNIDLV--------DVSSPSLPETSQRISYLSPHLCVRFHLPVKAF-   42
QQG34652.1_DiMV1         1   MVLFYFGLGNELASRRFFSPSPNFHLNHILLQAAAASHLAFSTLKPSDIKPFTTTPSSNIPP-LPRPTPI   69
YP_002756536.1_GSyV-1    1   MAAPAT-----------AYASPSAAFFALFQEQDLRCFR------PLTLAHSLRYDAPVRPRQLP-----   48
QUS93818.1_GRVFV         1   MAFPSNLSTM---LPAHLYQRPSHSLW--FNPSSPQPFEVLPHMVPANFPHLFRNIPTPLERSMVPRSL-   64
Consensus_aa:                Ms....................................................................
Consensus_ss:                                               eeeeeee


Conservation:
YP_009505639.1_BlVS          ----------------------------------------------------------------------
MZ422607.1_GaMV              ----------------------------------------------------------------------
ADD13602.1_OBDV              ----------------------------------------------------------------------
AYC35261.1_CSDaV        53   DAPIRCYRPLTYANHLRYDRSASSLKTSPVKLP-------------------------------------   85
QLM05437.1_GAMaV        51   ---------------------PSPISLTPEPSSLP-------------------QLPLSPAHLCRVLNHV   80
YP_009222597.1_NeVM          ----------------------------------------------------------------------
MZ305310.1_PGMV              ----------------------------------------------------------------------
QID59334.1_SbMV              ----------------------------------------------------------------------
QID59002.1_CaMaV        53   ---------------------PLTYRSYIRLVPAY-------------------GTPPKGLRPLLKKSGL   82
QQG34656.1_DiMV2        52   ---------------------YPASSALDAPLHHF-------------------RPLTLQRFHEQNWRTH   81
YP_004464920.1_SwMV          ----------------------------------------------------------------------
NP_115454.2_MRFV             ----------------------------------------------------------------------
QCC30252.1_PeDV              ----------------------------------------------------------------------
SBuMV-BU1                    ----------------------------------------------------------------------
YP_003475889.1_OLV-3    53   ---------------------TRAQMFSPTKAILSPAASPTPASQPDPPAEPCSDPPTETTVPSSKRALL  101
YP_009551972.1_AVF      51   ---------------------PHSEPIF------------------------------------------   57
ATJ00054.1_MsMV1        43   ---------------------PRKPQPTF-----------------------------------------   50
QQG34652.1_DiMV1        70   RKPKPVFIPPPPSTNVSRRHRRVSHAAYAHQTPHSTSPYLNAPLTFPSLNITATPLPLSRHSPNTYHPHA  139
YP_002756536.1_GSyV-1   49   ----------------------------RLRSITV--------------------PITSLDEGFTPILIA   70
QUS93818.1_GRVFV        65   ---------------------LGDFKPSRLRPVTLFGGAPPPLGPIIHPAYVAPEPLSSIPITFAP--AA  111
Consensus_aa:                ......................................................................
Consensus_ss:

                                                            MTase-GTase
                                                         CS
Conservation:                                              6 96      5      9 79 97897 68759  5 659
YP_009505639.1_BlVS     39   ------------------------------LQGA----GFKDVIEALAPTSHRDTISSALLETVVDPFRS   74
MZ422607.1_GaMV          1   ---------------------------------G----GLKEVIEFLTPTIHRDTITSPLLETIATPFRS   33
ADD13602.1_OBDV         19   ------------------------------VTGG----GLKDVIETLSSTIHRDTIAAPLMETLASPYRD   54
AYC35261.1_CSDaV        86   ------------------------------LTGG----TLADAILSLAPTTHRDTIATPLMEALAEPYRQ  121
QLM05437.1_GAMaV        81   R-----------------------------LTGG----GLMDVIQSLAPTLHRDTIASPLVEAIAEPYRR  117
YP_009222597.1_NeVM     29   ------------------------------LVGG----GLREVLAALAPTTHRDTVAAPLLEAVATPFRD   64
MZ305310.1_PGMV          3   -----------------------------------------KAISFLTPTNHRETITAPLLERIAAPFRG   31
QID59334.1_SbMV         20   ------------------------------FRGA----GFADAVAALTPTIHRDTITAPLLESLAPSFRT   55
QID59002.1_CaMaV        83   GRSLP--------LRDLARQLHAASHPRPYLRGG----GMRDAVDVLTPTTHRDTITAPLLESLAPTFRG  140
QQG34656.1_DiMV2        82   RIHLT--------GSSPNRVSFKSLPASPSLRGG----CMKDAIDSLTPTTHRDTITAPLVENLATPFRS  139
YP_004464920.1_SwMV      3   ----------------------------VFLRGG----HLLSGVESLTPTTHRDTITAPIVESLATPLRR   40
NP_115454.2_MRFV         3   ----------------------------SFLRGG----HLLSGVESLTPTTHRDTITAPIVESLATPLRR   40
QCC30252.1_PeDV          2   ---------------------------------------FKTIIDSLSSTAHRDTVSSPLVEAVSQPLRE   32
SBuMV-BU1               44   -------------PEQTPAQSQTSTQELPLLSGG----GMKDVLDFLTPTIHRDTVSSPIMEKIAAPLRS   96
YP_003475889.1_OLV-3   102   DSHSP--------SPVIPVDETAPISPPIRLTGS----GLKDLVDFLNPTVHRDTIAAPLLETISQPLRD  159
YP_009551972.1_AVF      58   ------------------------------LSGG---TLFQNAIDALSSTVHKDTITSPLLESITKPFRS   94
ATJ00054.1_MsMV1        51   ------------------------------LSGG---TLFQNAIDALSSTVHKDTITSPLLESLTKPFRS   87
QQG34652.1_DiMV1       140   KVHVPNLLFKPISTRKLIKQILRKSPPRIVLTGGAPSTPFRSIFEALSSTIHRDTVAAPLVEAVATPLRN  209
YP_002756536.1_GSyV-1   71   RPSLP-------------------------LLGG----GLKELVEMLAPTTHRDTVASPILEAVAGPLRT  111
QUS93818.1_GRVFV       112   EPKTP--------SPTAISRRFYRPS----LSGG----GLKELVEILNPTVHRDTVCSPLVEAAAGPLRD  165
Consensus_aa:                ..............................LpGG.....h.phlp.LssThHRDTlhtPLlEslt.PhRp
Consensus_ss:                                                      hhhhhhhhh  hhhhhhhhhhhhhhhhhhhhh
```

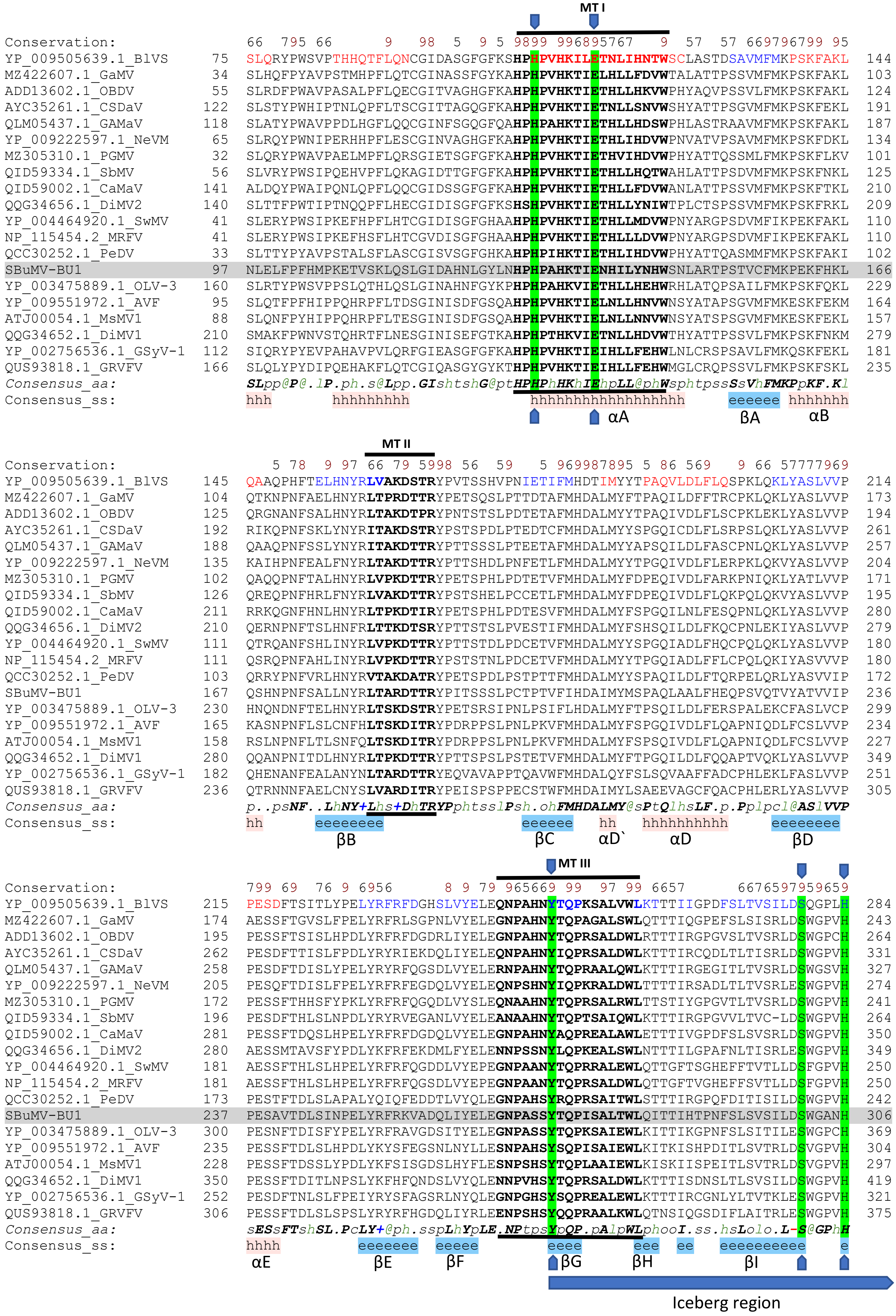

MT I
MT II
MT III
Conservation:
Consensus_aa:
Consensus_ss:
αA
βA
αB
βB
βC
αD`
αD
βD
αE
βE
βF
βG
βH
βI
Iceberg region

```
Conservation:                     7798677 56 5   7   9  9 96 79 7 67 9   979989  78 558 97969999958995877
YP_009505639.1_BlVS        285    SLLIQRGEPPLHSRHDTAEFRTPPAILLPAPSSLNQDVRHRLVPKKVYDAVFLYVRTVRTLRVTDPAGFI    354
MZ422607.1_GaMV            244    HLLIQRGLPPVHARHDELDFRVPAAILLPEASSLHQPIRDRLAPKAVYEAIFIYVRAVRTLRTTDPAGFV    313
ADD13602.1_OBDV            265    SLLIQRGIPPMHAEHDSISFRGPRAVAIPEPSSLHQDLRHRLVPEDVYNALFLYVRAVRTLRVTDPAGFV    334
AYC35261.1_CSDaV           332    SLLIQRGKPPIHLEEDSLSFRAPKAVLLPEPSSLSQAVRDRLVPADVYQALFIYVRAVRTLRVTDPAGFV    401
QLM05437.1_GAMaV           328    SLLIQRGTPPVHAERDTISFRAPKAVVLPNPSSLTQPLRDRLVPKDVYTAIFVYVRAVRTLRVTDPAGFV    397
YP_009222597.1_NeVM        275    SLLIQRGVPPVHQEVDTVSFKAPRAVALPEPSSLHQDLRHRLVPKDVYDALFIYVRAVRTLRVTDPAGFV    344
MZ305310.1_PGMV            242    SLLIQRGKPPIHVDDDTVEFTTPEAYALPQPASLHQDLRHRLVPAKVYNELFVYVRAVRTLRTTDPAGFV    311
QID59334.1_SbMV            265    SLLIQRGVPPHHAERDSVSFTGPDAILLPEPASLTQDPRHRLVPSKVYHELFVYVRAVRTLRVTDPAGFV    334
QID59002.1_CaMaV           351    SMLIQRGEAPLHQRYSRASFRGPEAVCLPSPASLHQDLRHRLVPKKVYDAIFMYVRAVRTLRVTDPAGFV    420
QQG34656.1_DiMV2           350    HLLIQRGEPPVHSREDLLDFKVPQAILLPAPSSLNQEIRHRLVPKKVYDSLFVYVRAVRTLRVTDPAGFV    419
YP_004464920.1_SwMV        251    SLLIQRGRPPVFSAEDSVSFRVPDAIALPAPASLHQDLRHRLVPRRVYDALFNYVRAVRTLRVTDPADFV    320
NP_115454.2_MRFV           251    SLLIQRGRPPVFQAEDIASFRVPDAVALPAPASLHQDLRHRLVPRKVYDALFNYVRAVRTLRVTDPAGFV    320
QCC30252.1_PeDV            243    SLLLQRGRPHMHEDEDTVYFKVPKAVALPEPASLRQDLRHRLVPADVHDALFVYTRAVRTLRVTDPAGFV    312
SBuMV-BU1                  307    SLLIQRGLPPVHHSHDTHSFKVPDAILLPEPAHLNQPIRHRLVPTAVYQAVLLYVRAVRTLRTTDPAGFV    376
YP_003475889.1_OLV-3       370    SLLIQRGLLPLHQEHSTQAFKTPEAILLPAPSHLQQDLRHRLVPTRVYESIFVYVRAVRTLRVTDPSGYV    439
YP_009551972.1_AVF         305    SMLISKSNFPFDAETDTISFKVPSAILLPEPNSLSQDPRHRLVPTKIYHAIFAYVRAVRTLRVTDPAGFI    374
ATJ00054.1_MsMV1           298    SMLITKSQLPFDASQDRIDFKVPSAILLPEPNSLNQNPRHRLVPTKIYHAIFAYVRAVRTLRVTDPAGFI    367
QQG34652.1_DiMV1           420    SLLIQRGLAPITSSHDTITFRTPSAILLPEANSIRQDIRHRLVPKDIYHNLFSYTRSVRTLRVTDPAGYI    489
YP_002756536.1_GSyV-1      322    SLLIQRGKPSVHLEHDEVSFVGPDAVALPEAAALRQDLRHRLVPRTVYDALFVYVRAVRTLRTTDPVGFV    391
QUS93818.1_GRVFV           376    SLLITRGRPPV-LTDDSLFFSSPDAVEIPAASSIQQSLRHRLVPSAVYHSLFIYVRAVRTLRQTDPQGYV    444
Consensus_aa:                     SLLIQRG.sPhc.ppDphsFpsPpAlhLP.ssSLpQshRHRLVPppVYptlFhYVRAVRTLRhTDPtGFV
Consensus_ss:                     eeeee                   eeeee               hhhhhhhhhhhhhh      hhhhh
                                   βJ                       βK                    αF              αG
```

```
Conservation:                     999 59695 999669999977967 95 695    5  6 9   6 58    6   5
YP_009505639.1_BlVS        355    RTQSSKAEHSWVTSFAWDNLAHFALQTASHRPRTTYYLFSSALARLSHWCRNHQLALSWASVVAISPAAA    424
MZ422607.1_GaMV            314    RTQSNKPEHSWVTASAWDNLAHFALMTASHRPHTTYYLFDSTLSKVAHWARTHTLFLQGITAACLTPLAS    383
ADD13602.1_OBDV            335    RTQCSKSEYAWVTSSAWDNLAHFALLTAPHRPRTSFYLFSSTFQRLEHWVRHHTFLLAGLTTAFALPPSA    404
AYC35261.1_CSDaV           402    RTQISKPEYSWVTSSAWDNLAHFALATAPHRPHTTYFLFNSTAARVAHWFRTHTLALLSGATAAAAGLLM    471
QLM05437.1_GAMaV           398    RTQCSKPEYSWVTASAWDNLAHFALSTAPHRPRTTYFLFHSLHARLSHWVHHHTLALTTVGACAASVLAF    467
YP_009222597.1_NeVM        345    RTQCSKPEYAWVTSSAWDNLAHFALSTSPHRPKTTYILFHSSFDKVVHWLKTHKLLMTSAGASLLSVASS    414
MZ305310.1_PGMV            312    RTQCSKPEYDWVTSAAWDNLQNFALKTAAVRPGFDHLMFKSLWAQVDHWCRHHTLALLTAATPFAALACA    381
QID59334.1_SbMV            335    RTQCSKPEYRWVTSAAWDNLAHFALLTAGVRSGTTHPMFQSLFARIHHWVRNHHLVLLSLATPATSLATA    404
QID59002.1_CaMaV           421    RTQCSKPEYSWVTSSAWDNLQHFALTTASHRPRTTFHLFTSAFARCTHWIMTHGLGIRTAVTLPIAGLSG    490
QQG34656.1_DiMV2           420    RTQCTKPEFAWVTSSAWDNLAHFALSTANHRPRTTFFLFDSTFSRISHWVKTHTLTLGWFSAFLATPLIN    489
YP_004464920.1_SwMV        321    RTQVSKPEYSWVTSSAWDNLQHFALQTAAVRPNTSHPLFQSPLARVAHWLRTHTWALWCLASPAASLTAW    390
NP_115454.2_MRFV           321    RTQVGKPEYSWVTSSAWDNLQHFALQTAAVRPNTSHPLFQSPFARLSHWLRTHTWALWCLASPSASVSAW    390
QCC30252.1_PeDV            313    RTQCSKPEYSWVTSAAWDNLQHFALQTAPHRPHTTFYIFPSMFAKLRHWMRAHTKALQLSGIAAASLVAS    382
SBuMV-BU1                  377    RTQQSKAEYSWVTSAAWDNLAHFALLTCSHRPNSRYFLYSSSFSRLVGWIHAHRLALLTISTPPSSALLG    446
YP_003475889.1_OLV-3       440    RTQSSKPEYSWVTTAAWDNLANFALLTCAHRPRARYYLYWSTLDRLMGWIRAHKLALLTASTPLASLGLA    509
YP_009551972.1_AVF         375    RTQASKPEFSWVTSAAWDNLQHFALHTSPHRPISHYMLFRSPLQRFQHWARTNSYYFYLASCGIALPFVS    444
ATJ00054.1_MsMV1           368    RTQASKPEYSWVTSAAWDNLQHFALHTSPHRPISHYMLFRSPLQRFQHWARTNSYFFYLASCGIAAPVLS    437
QQG34652.1_DiMV1           490    RTQASKPEFDWVTSAAWDNLQHFSLHTSPHRPVSHYMLFRSPFQRLKHWATTNEYHIFTLLALLATPIAF    559
YP_002756536.1_GSyV-1      392    RTQSNKAEYSWVTSAAWDNLQHFVTETAAHRVPNRHFFFNSTFAKCRYWCSQHKLGLLTVTTPPACGLTL    461
QUS93818.1_GRVFV           445    RTQSNKPEYAWVTSAAWDNLQHFVTETAHHRQRTTYYLFNSTLARLSHWCRCHKLAIATSLTPLASASIL    514
Consensus_aa:                     RTQtsKPE@tWVTStAWDNL.HFtL.TtshRP.sp@.hFpS.h.+h.@Whphhphhl...sh.hhs.hh.
Consensus_ss:                     hh           hhhhhhhhhhhhhh         hhhhhhhhhh  hhhhhhhhh     eeee hhhh
                                                  αH                     αI          αJ          βL
                                                                                    TMD
```

```
Conservation:                                     5           7                      6
YP_009505639.1_BlVS        425    LSSLSVSKFLRSELNSLAIFRRWI--KAPP-----HF-L-----FAPKAPFLSMSFRALRTGPLLFANTG    481
MZ422607.1_GaMV            384    LSALGLAHFSRCRISALSLFGHWL--VCPD------FYL-----LKPKAPLFRLAVAPASAPFRAFKGTF    440
ADD13602.1_OBDV            405    WLANLVARTSASHIQGLALARRWL--ITPP------HLF-----RPPSPPSFALLLQRNSTGPILLRGSR    461
AYC35261.1_CSDaV           472    TASWGFRAMISSHLVSLSICKRWL--RAPP------HLL-----WPEKAPWFQLTLRPKVTGPLIDLPIL    528
QLM05437.1_GAMaV           468    VGCRVAAGLSNQQLRSLACLRRWI--VAPP-----HLPF-----FRPKAPLLELTLRPKVTGPLVNLPVL    525
YP_009222597.1_NeVM        415    SLFLAIGRVLNSHVHSFAVFRRWL--KPPP------HLL-----WPPKAPLLELSLRARSTGPTVLSGTP    471
MZ305310.1_PGMV            382    TIVKAGETLYHSHLDELVVARHRLVGSQPF-----RLSI-----WKERLPLFRIKMHRECTGWTFFQGSI    441
QID59334.1_SbMV            405    TGVSALGHLAHRHIDELTVAGRTLIGRAPR-----HFSI-----WKPATTWFRLRTHAPCTGWTLWPGTI    464
QID59002.1_CaMaV           491    ASCWLFHRLITGHVDQCAILGRWV--KVPF-----GMSKLPRI-ALPPAPWFSLQTHAGGAGIKLLRGTR    552
QQG34656.1_DiMV2           490    RLACSLSNFTCSKVTSLSLLKFWI--IKPK------YFF-----RLPPARLFSLTX--------LFKDTI    538
YP_004464920.1_SwMV        391    LAASRFGRLTVLHTDRLRLFGCDL--IGRR--------FWPILPFQSPEARFLWETHSACKPYTLFAGSA    450
NP_115454.2_MRFV           391    ATASALGRLLPLHTDRLRLFGFDI--IGRR--------FWPRLPFHGPEPRFLWETHPACRPPVLFADSA    450
QCC30252.1_PeDV            383    TAAYATARIARCPIESLAVCGWWI--KSPP---TIKHALREAVGFPQKAPQFSIALTPRPAAPFGWK---    444
SBuMV-BU1                  447    LTSFSLARFYWRQISTLVLFRFPI--ISPPTSFLSRLSF-----RSALPPLFKLQTVPRPVPW-------    502
YP_003475889.1_OLV-3       510    SSALLACRPFWMRLDSLQLLNRWV--IKPS--FPRNLPFLLPKAFQPKASLFKAEFLPRHAPS-------    568
YP_009551972.1_AVF         445    ALSLTLSHLSRCSVHHFSLFRHWF--VPPP-NPHFLFPNFLHSFLLPKAPFFSLQLHNPHPFPTLSK---    508
ATJ00054.1_MsMV1           438    LSSVLAHHYSRCSILHLALFKKWI--VAPP--SSNFLPFFLSSRFIPKAPFFSLALHNPVPRPTLRF---    500
QQG34652.1_DiMV1           560    KLSSFLTTAPRLYIKQLTVFSHHL--VGRPSLAHRYLPPIIRH-FLTPTPRFSLHLGTRPAI--------    618
YP_002756536.1_GSyV-1      462    FTGAKLASAMSSRLTALAVFHHWV--VPPP-TLF----------FTPKAPLLAIQLTRLPQP--LFSSVP    516
QUS93818.1_GRVFV           515    LALGGVDLLWKHSIKHLVIAHRYLIGSAPL-----HARLLPRFLVQQHYPRFSLALEPRVAAPVIHL--P    577
Consensus_aa:                     h.t..h..h...plpphslh..hl...sP.......h.h.....h.s.hs.hpl.h.s.....hl.....
Consensus_ss:                     h              eeeeeee                           hhhhhhhhhh
```

```
Conservation:                               6    65                     8       8 6 896 99 759 9
YP_009505639.1_BlVS     482   FQTRLFPTAAQNFCSANPLLASFF-PVKPLHRG-AFLASLLAASVPVALLLV-RQFVGPDSPQSMHDAYA   548
MZ422607.1_GaMV         441   LAFDVFPRLTQDLLHRVPLLRRFF-PRRPLPNA-CWWACLAAASIPAFLWAC-RWFVGPDSPQHMHDAYH   507
ADD13602.1_OBDV         462   LEFEAFPSLAPQLARRFPFLARLL-PQKPINPW-IVASLAVAVAIPAASLAV-RWFFGPDTPQAMHDRYH   528
AYC35261.1_CSDaV        529   RPFRLFPSTCAKLGAKHPALATLL-PAAPRPTW-PLKVGLALAAVPVCLFLW-RNFIGPDSPQDMHDSYH   595
QLM05437.1_GAMaV        526   RPFRLFPTTCARLGARHPAVALFL-PPKALTSS-WLPYALGAAAIPLALLGI-RWFLGPDSPQAMHDQYH   592
YP_009222597.1_NeVM     472   FAFKLLPKTFCRLGASYPSLAKFL-PKCPIPRK-YLWLSIAAASLPLIALGV-RFFLGPDSPQSLHDRYH   538
MZ305310.1_PGMV         442   FETRPFDNLCRSLG-KKPAFARFL-PTRPIGGK-WLWAMAAVAAVPAALLAI-RWVLGPDSPQALHDRYQ   507
QID59334.1_SbMV         465   FETKILHRVAADLG-RSRWLARYL-PNRPLHRY-WTYAALGLSAVPAIALAI-RWFLGPDSPQALHDRYQ   530
QID59002.1_CaMaV        553   FEFQLFNQLFRSWA-RNPWLAQFL-PRRRMGKL-GWSFLLTSIMAPILSLAA-RKFLGPDSPQSLHDSYH   618
QQG34656.1_DiMV2        539   LQCQPFQNFCQQLAIKFPPAAHFL-PQKPLPFI-ALFMCVASTLAPTVVMGL-RQFFGPDSPQAMHDSYH   605
YP_004464920.1_SwMV     451   FECRVLGALAHRCS-PSPTLSRLF-PEAQPARW-VVFGSLALAAVPLTALLV-RWFLGPDSPQALHDQYH   516
NP_115454.2_MRFV        451   FECQILAGLANRCS-PSPFWSRLF-PTASPPSW-VAYSALALAAVPLAALAL-RWFYGPDSPQALHDQYH   516
QCC30252.1_PeDV         445   --------LLLKPLKQAPWLRPLY-RDAPVPRF-WIKATLAALAAAAAAAAL-RHFLGPDTPQHMHDKYV   503
SBuMV-BU1               503   ---RLLPSLSMSLLSKFPVLRRFF-SDKPIPSW-FYYVLCSVSSIPLLLLSL-RKFCGPDSPQALLDNYT   566
YP_003475889.1_OLV-3    569   ---RILPEFFDALLKKAPFLRPLF-SDAPIPSW-AWALCATGVAIPIIALSI-RRFLGPNSPQALLDEYQ   632
YP_009551972.1_AVF      509   ---VLSFLLPTSFINQLKFL-----QPRSLPKH-TLHCTLALASLPILAALY-RWFSGPDTPQALHDQYE   568
ATJ00054.1_MsMV1        501   ---ILSKFFPESFLSRFPSL-----NPRNLPKS-TVPVALLLAAIPAFAALY-RWFSGPDTPQALHDQYE   560
QQG34652.1_DiMV1        619   ---HPDFNILKPLLHKIPWLISLL-PKAPTPLA-ATLFIASMSAIPLAFWCH-RRFFGPDHPQALHDQYQ   682
YP_002756536.1_GSyV-1   517   FLHKPLGKLSLRLLNRFPFLRRFF-PDAPIPTW-ARLLTVAIALSPAVWLAI-RHFIGPDAPQALNDHYV   583
QUS93818.1_GRVFV        578   FSQTVRLTFLTTLANHIPSLKRFL-PRLSTPAL-LKAAFCLGLLTPPLLLLI-RSFVGPDSPQSMSDAYN   644
Consensus_aa:                 ...p....h..ph..p.P.L.phh.s..shs...hh.h.hh.h.hPhhhh.h.R.FhGPDoPQthhDpYp
Consensus_ss:                                  hhhhhh         h hhhhhhhhhhhhhhhhhh hhh     hhhhhhhhh

Conservation:                   896  9 9   7           8 5                     8
YP_009505639.1_BlVS     549   DLFHPADWRLIFNRKPLFASPEP--FLPIT-------------TVPA--PVD------------------   583
MZ422607.1_GaMV         508   ELFHPELWRLTLRRSPLAVSAER--FLPIETFTPPE------PTPPT--PAQEATAAETPVL--------   559
ADD13602.1_OBDV         529   TMFHPREWRLTLPRGPISCGRSS--FSPLPHPPSP---------TPA--PDSRAGPLQPPSALPS-----   580
AYC35261.1_CSDaV        596   AMFHPQPWGLTLTRKAICCDRAP--FLPIPVVPSS--------DFKA--PPTPATPLLTSIPIKGVEPQV   653
QLM05437.1_GAMaV        593   AMFHPPDWHLTLDRAPLLTHRQP--FLSFEPQPAP--------EELL--PPTAAVRLETPPAPPTSAQ--   648
YP_009222597.1_NeVM     539   ALFHPEPWELVLKRGPVHVARAP--FLPFTP------------VDHV--PLDSD----------------   576
MZ305310.1_PGMV         508   ATFHADPWRLILHRGPITVGRRS--FLPLQDL-----------DTPK--PQPPDSP--------------   548
QID59334.1_SbMV         531   ATFHPDPWQLRLXRGPITCGRRA--FLPLEHA-----------------PPASPSP--------------   567
QID59002.1_CaMaV        619   AMFHPEPWTLTLDQGPVFCTSTP--FLPVP-------------TVPT--PAAASFPDN------------   659
QQG34656.1_DiMV2        606   RMFHPELWHLTIERKSRYSNPAQ--FLPLDFFPQPEPVTSKAIVSQA--PSSDVSRPLSRLPEVQTP---   668
YP_004464920.1_SwMV     517   ATFHPDSWSLDLPRRLRSCVREP--FLL---------------TGAA--PASTPTSAPENFSPD------   561
NP_115454.2_MRFV        517   ATFHPDPWTLDLPRRLRRFERES--FMR---------------TGSA--PLPLSLP--------------   553
QCC30252.1_PeDV         504   ALFHPQPWQLKIKRGVIKTLPAP--FLPFE---------------PT--PPI------------------   536
SBuMV-BU1               567   DFFHSKPWTLTLDRAPVHVRPSS--FIPIESLPPQAPQPLYGNSDHLFHPAETPTIVPISNPDSEPF---   631
YP_003475889.1_OLV-3    633   SYFHPKPWQLILEQGPIFANPEP--FSPH--------------CQPI--PSSGPDSAPSSTLGTPPM---   681
YP_009551972.1_AVF      569   TYFHPKEWTLSFPRKAITVTRIP--FLP---------------TDPI--PISDA----------------   603
ATJ00054.1_MsMV1        561   EYFHPKEWSLFFQRKPVHVTRSP--FLP---------------QDPI--PVSNA----------------   595
QQG34652.1_DiMV1        683   AYFHPKDWTLTLDRAPLTTSPQP--FLPF-----------------------------------------   709
YP_002756536.1_GSyV-1   584   RFFHPDRWQLTFERQPRFVALDR--TFPWPLPQAPE------PTEPR--DSDVPLETVP-----------   632
QUS93818.1_GRVFV        645   RYFHPSRWELSLERSPASVIPSPPAFLPTASAAEP--------TEPR--PSDSPLE--------------   690
Consensus_aa:                 .hFHPp.WpL.hpR.sh.h...s..FlPh..............s..h..Ps.ss................
Consensus_ss:                 hh      eeeeeeeeeee

Conservation:                                       9                                   5
YP_009505639.1_BlVS     584   ---------------------LPQIE----SIAIP----------------------AP----SPAPPSP   602
MZ422607.1_GaMV         560   ---------------------APAVP----ASTAP----------------------SPPSPLPASEPVQ   582
ADD13602.1_OBDV         581   -------------------THEPAPA----DLESP----------------------APQAHAPQTEPPS   605
AYC35261.1_CSDaV        654   SGEGVPPQSASSTGPASDSRRAPQPA----SSTGP----------------------DPPTQNTSAAPQP   697
QLM05437.1_GAMaV        649   -----------------VEPPTPAEP----KSTRP----------------------APLKPVLVATPPP   675
YP_009222597.1_NeVM     577   ---------------------SPAIP----APPVP----------------------QPQAPLSPVTPAP   599
MZ305310.1_PGMV         549   ---------------------GPPPP----SPAGE----------------------SESASFTPEKPAP   571
QID59334.1_SbMV         568   ----------------------PSPA----DKPAT----------------------SPVHVQTPATPPP   589
QID59002.1_CaMaV        660   ---------------------PPLPV----SEVSP---------------------QIPLQAVSPPSPPQ   683
QQG34656.1_DiMV2        669   ------------------SESQPKEVAPSNLTQMP----------------------QSQKLSSPAEPAP   698
YP_004464920.1_SwMV     562   ---------------------TPRPLTSLRAQA------------------------EPSQPSDVQPPKP   586
NP_115454.2_MRFV        554   ---------------------PPEGSLLP-VEPPL----------------------APSDPEPALEPSP   579
QCC30252.1_PeDV         537   ---------------------LPSD-----SSDEE----------------------VEERFQPKLAPIP   558
SBuMV-BU1               632   ------------------PPPTPIPELPPFSPKKP----------------------VPSVSKPPTSQEP   661
YP_003475889.1_OLV-3    682   -----------------YPSSPPAPVE---EKTIL----------------------VPTVRALPPAPPP   709
YP_009551972.1_AVF      604   ---------------------HPEPS----ESE------------------------IPLFNSTPNEHSP   624
ATJ00054.1_MsMV1        596   ---------------------HPDPS----ESD------------------------IPLEPSSSPLTPP   616
QQG34652.1_DiMV1        710   ---------------------EPIPT----QPESL----------------------NIPSNATPASPAP   732
YP_002756536.1_GSyV-1   633   ---------------------SPLPV----VAPLP----------------------APATSVPPVDTSA   655
QUS93818.1_GRVFV        691   ---------------------VPL---------------------------------VPSPRLHPDTPPP   706
Consensus_aa:                 ......................P.ss................................s....ss..sss
Consensus_ss:                                               eee
```

```
Conservation:                        6                         5                                        8
YP_009505639.1_BlVS     603  TS------LAPTPIPPVTSTLDPAPASASAPAA------------------------------TPPAIAA  636
MZ422607.1_GaMV         583  ALQN----ASPAPAP---PAQLTSLSSLPTPAP---------------------------RDTIFPLASTA  619
ADD13602.1_OBDV         606  PVIEQE--ARPDPFP--APA----------PRP----------------------------APTP-SASA  632
AYC35261.1_CSDaV        698  PIESKVTFAQPIESV--APVVPG-AGEP--PQSASSTGP-----ASVSRRDPQVASSTTPDAPTLDVSVT  757
QLM05437.1_GAMaV        676  PVTAPT--SQPEPTQ--APAAPT-LAIPASPRALQAEADRIHSVQMADRTPTEIAIVAAADALLPLAAST  740
YP_009222597.1_NeVM     600  VSRDSPT-SDPPGRDAEEKAVNAVLSLPSPPSQ---------------------------------PPVR  635
MZ305310.1_PGMV         572  AATQPQAQAPPVATPAPAEAIEK-DHPPSVPAA---------------------------------AAEA  607
QID59334.1_SbMV         590  P---------------------------------------------------------------------  590
QID59002.1_CaMaV        684  PDSSPT--PEPVPVPASTPPPPPELSEPAPPPA----------------------------VEAPQAPAP  723
QQG34656.1_DiMV2        699  KTSTE---PAPGPVPTIESAVQS-IARALNPDAVHNPDP------VNALKDINSTAEAVRDLLSPICPPS  758
YP_004464920.1_SwMV     587  SAVQPQVAPEPAPAP-ISPEPAASLPISAEPES----------------------------VFEPPTSHSP  628
NP_115454.2_MRFV        580  PAA-----SVPAPAP--APA--------SEPPP--------------------------------SPESVAP  604
QCC30252.1_PeDV         559  PV--------PQPAP--STATPPPQPAATAPPEP----------------------AKPVAQQLIPAAPVE  596
SBuMV-BU1               662  AVSNPPAPSSPQPPSTPSTPRYLPPHKASEPKP---------------------------LQVHPLPPPS  704
YP_003475889.1_OLV-3    710  SS------VPPPSKP--SPAAPAPVTPPSLPTP----------------------------VIPPLSTPT  743
YP_009551972.1_AVF      625  S-------SSPEPSP--LPAVPPPSAIPPVQLP-------------------TVASNNPGLDPKRPSSSLL  667
ATJ00054.1_MsMV1        617  --------ESPESSPPLAPTVPPPSAIPTVKLP-------------------STSSHNPGLDKSKPAPSPL  660
QQG34652.1_DiMV1        733  TTQAQEHASTPLDTP--SKPPNPPTSSPPTPPP---------------------CPTSDFVSSDRPHQSPL  780
YP_002756536.1_GSyV-1   656  TTVSAVEPSLSTESL--KTVEAPSGTTILQPRE--------------------------LKDTIFPLPAAA  698
QUS93818.1_GRVFV        707  AE------KQPEPTPC-VVASPAPVLDASAPRA----------------------------EETPAGLDM  741
Consensus_aa:                ss........P.sss....s..s....s..P.s...........................P.sss.
Consensus_ss:
```

```
Conservation:                                                6    6
YP_009505639.1_BlVS     637  PA---------------------------PAPEPQAEALSL----------------LIPSEHQPAAPPPIP  665
MZ422607.1_GaMV         620  PIAELTAPTA-------------------PVAEPSAPAPTDGR--PEDFAPPLDPSTVDAALLALGISIPSS  670
ADD13602.1_OBDV         633  PS---------------------------PAPTPSAPEPPS------PTASE----------QAASLIPA--  659
AYC35261.1_CSDaV        758  PPKTIYPIDHLQNDFGPCRCSVCEPLQPAPVPSTPLTVS------DHKEAQDAEALSSALQALGLAPT--  819
QLM05437.1_GAMaV        741  PAADSAPE---------------------PTVVPGPPEEVD------PAGQA--------ALASLGLAPSLP  777
YP_009222597.1_NeVM     636  P----------------------------PSPSVQAPASSD------DLADRRDAENLAQALSSLGLFTPDP  673
MZ305310.1_PGMV         608  P----------------------------PSARPVEPPSET------QKQESQRPSPSEEAAMLCGERAP--  643
QID59334.1_SbMV         591  -----------------------------PSPEPASPSEPD-------------------------AADIPTP  609
QID59002.1_CaMaV        724  PAD--------------------------PSDVPAQPAPVE--------FSFPDPPAETHVASMFGVPTPLD  761
QQG34656.1_DiMV2        759  LASHHQVA---------------------PASAPAIKTQPP-------TPSQSSDNLLQTAMLSTAEEVKLD  802
YP_004464920.1_SwMV     629  PAQPVADSE--------------------PTSQPSSRAPTP----------VRQAALLGADLRFGDLPPRSS  670
NP_115454.2_MRFV        605  PVAVVA-----------------------PAVQPARAPSPS-------------PALLGAELRFGDLPPVSA  640
QCC30252.1_PeDV         597  SQETVDAIKRQLADPM---------PALPAEGPSSPRPLSNALLCPPEGSEVLLEAPPADCAVTASPPPGF  658
SBuMV-BU1               705  PPVRIPQVGASGFV---------------AQDLPAQELPLP------------TLSCELVDLDQQPSTPPSP  749
YP_003475889.1_OLV-3    744  PETRVPEVGKSGIV---------------ASDLPPTEISPN------PTLARCRPNFLGLCAPHPNSNPSHP  794
YP_009551972.1_AVF      668  TAIIDPSTNA-------------------PATSSSLPPATT-------NPTPVQSEPFDPFSFSAPQTAPRI  713
ATJ00054.1_MsMV1        661  TSIIDPSTNEPLLKQP--------SLQPVSVPTDPFDFT--------------------------TPSFP  696
QQG34652.1_DiMV1        781  DQFPVPPTD--------------------PQGKPAPPSTNEHL-FRHPLPKAPDALPENLFPDFFNQPAPPR  831
YP_002756536.1_GSyV-1   699  LAVTPPE----------------------PAPAPAEPVSAS--------------------------------TV  719
QUS93818.1_GRVFV        742  SATTV------------------------LEAKPPVQVSLV-------------------------DEPPRP  764
Consensus_aa:                s..........................P.s.Ps...............................s..ss..
Consensus_ss:                                                       eee
```

PRO[1]

```
Conservation:                     5                          5
YP_009505639.1_BlVS     666  -ELLPAAG-----------QLQLE---SSGVVSNL--------------------TPAAPEPA-APEPSP  699
MZ422607.1_GaMV         671  DIVLPPSP-----------AAAPEPIMPPNSLVQS-----------------SFGSVAAPVSS-LPAPTP  711
ADD13602.1_OBDV         660  ----PSS------------ALVVE---PSGVVSAS-----------------SWGATNQPAD--QVDDSP  691
AYC35261.1_CSDaV        820  -PTAPQSQ-----------NLTVE---SSGAMHAS-----------------SWDQLSSPSS--DWDPSP  855
QLM05437.1_GAMaV        778  ASTGPSQ------------SANLE---PSGAVHAA-----------------TWDQSSAPSS--DWDTSG  813
YP_009222597.1_NeVM     674  -HSEPQQP-----------VVG-----SSGELHAR-----------------EWGQEDAPAS--DPESSP  707
MZ305310.1_PGMV         644  ---SPPNP-----------LLTVE---PTGEVYSR--------------------EPAGPVTE-DPERVA  675
QID59334.1_SbMV         610  VPDAPART-----------PVVDE---KTAIISDP--------------------EPPEPPKP-DPECSE  644
QID59002.1_CaMaV        762  GKPAAPNT-----------PLTVE---ERGHVYTT----------------------APLPEPETPAASDP  796
QQG34656.1_DiMV2        803  QHLES--------------NLHLE---PKGHVFSS----------------------EPSWADE-VEGLSP  833
YP_004464920.1_SwMV     671  WAQDPELS-----------PLGES---TQGTVFAQ----------------------IPTPREP-ESALAR  704
NP_115454.2_MRFV        641  WDSDPEIS-----------KLGES---TQGTVFAV----------------------TPGPRAP-EPDTAR  674
QCC30252.1_PeDV         659  TSSAAPSA-----------PSQPE---APAQIEAFSSLLYGQDSISVQRDGSVVGAHTTPEPL-SEEVSA  713
SBuMV-BU1               750  PPASPS-------------PLVTD---DLGTVSAS---------------------RPKLDPT-ETTFSE  781
YP_003475889.1_OLV-3    795  EPSAPS-------------PLITD---PSGSLTAI---------------------NPTLEPF-EFNHAL  826
YP_009551972.1_AVF      714  VPACPPGF-----------ENILSD---KFGALNSS---------------------TAPAEPHSEPETSP  749
ATJ00054.1_MsMV1        697  APRPPPGF-----------ERVVSD---KHGVITES---------------------NLPHEPESHVEVSP  732
QQG34652.1_DiMV1        832  SSSAPPASVNLRPQTPFVKPIQPQD-CETGNIIPP-----------------PEVLIPLAKPD-PPPASP  882
YP_002756536.1_GSyV-1   720  LGTAPLSR-----------DL------HTGHVSTP---------------------ATEPGLV-EPEHSP  750
QUS93818.1_GRVFV        765  ESLLNPSAT----------QEELLIS---HTGTLTHT---------------------TPQPVTA-TPEVEP  801
Consensus_aa:                ....s..............h..p.....t.l.s.....................s.s........t.
Consensus_ss:
```

PRO I

```
Conservation:                    9  9 77 865   6   6 8    7    56 9   9 9     6    69    6999 66   66  6       98   8
YP_009505639.1_BlVS      700  LQADSSARGPVQLFSELFPGS-YIGTTGAFNSRYRASGRAPTPYPAGTDCLLVTIEQATSISRRDLWETL  768
MZ422607.1_GaMV          712  LESDPTAAGPISMFSELFPAS-YLGTSGSFLCRRRASGRCSLPYPA-MDCLLVTVSEATHIPREDLWSAL  779
ADD13602.1_OBDV          692  LARDPSASGPVRFYRDLFPAN-YAGDSGTFDFRARASGRSPTPYPA-MDCLLVATEQATRISREALWDCL  759
AYC35261.1_CSDaV         856  LARDSSASGPPGMYSDLFPAP-YLPGTGQFIFRSRANGRANIPYPD-MDCLLLSIEQATRLPKEALWDTL  923
QLM05437.1_GAMaV         814  LGRDGSANGPIALYSELHPAN-YLPSTGDFLFRDRANGRANLPYPT-MDCLLVAVEQATRIPKEALWDTL  881
YP_009222597.1_NeVM      708  LLRDPSACGPVAMYSELHPGN-YVPGTGLFQFRDRASGRAPVPYPS-MDCLLVAVEQATRLPKEALWDTL  775
MZ305310.1_PGMV          676  LQIDPTADGPIGLYSELHPDC-YEPRTGDFLARNRNSSRSDLEYPSGVDCLLKAVAQATNISVQALWTTL  744
QID59334.1_SbMV          645  LERDPSAAGPALPYDELYPDC-YTELSAKFLSRARNSALSAAPYPAGVDCLLTAVSEATGLQTQALWECL  713
QID59002.1_CaMaV         797  LGSDPSASGPIAFFRELYPAS-WLQNSGLFLCRSRNSSRSSHPYPS-NDCLLAAVSEATSIPRETLWETL  864
QQG34656.1_DiMV2         834  LQSDGSARGPVAFFSQVHPGS-YIGTTGQFNFRARASPSSKQSYPVNTDCLLRAVEAGSGISRQALWECL  902
YP_004464920.1_SwMV      705  LEVDPTASGPIMEFRDLQPGL-YLGTTGSFLTRARNSVSSTIQYPARAACLLVAVRNATSLPTATLWAAL  773
NP_115454.2_MRFV         675  LDADPSASGPVMEFRELQKGA-YIEPTGAFLTRARNSVSSSIPYPTRAACLLVAVSQATGLPTRTLWAAL  743
QCC30252.1_PeDV          714  LVRDPSAAGPVLQFHDLHPGA-YLEATAAFPCRLRNGQPSNMPYPQ-NDCLLRTVHTATHIPLAALRECL  781
SBuMV-BU1                782  LVADPSASGPVSSFSELYPNQ-WCPGSATFPARVRNSSHSTSPYPG-MDCLLKAISIPTGLSTKSLWESL  849
YP_003475889.1_OLV-3     827  LGADPSGQGPVAQFNELFPSTLWLQGTGEFLTRSRVQAPSSAPYPA-MDCLLHAIHTATRIPKPTLWAGL  895
YP_009551972.1_AVF       750  LLADQSAQGPVVMFGELYPAE-YHSNCDEFQTRARVNHSSNLPRPVINDCLLEAVSSATNISKDALWNAL  818
ATJ00054.1_MsMV1         733  LMADSSAEGPVVLFGELYPAE-YHANCDEFQTRRRVDHSSSLPRPSVNDCLLEAVSSATNITKDALWNAL  801
QQG34652.1_DiMV1         883  LLADPTATSDVKLWSEHLPRE-YLSNCGSFLFRERSTPASDLPYPSTTDCLLQAVSKATGIDPHALWRTL  951
YP_002756536.1_GSyV-1    751  LAADSSATGEVSEFFNLHPAD-WIAPTATFLARRRGETISGAKYPA-MDCLLAAVSAGANIPKDALWKTI  818
QUS93818.1_GRVFV         802  LMVDASGAGPVDSFSVLHPGS-YLPFSGSFLSRIRVSSLSPAPYPA-LDCLLVSVAGATGFPKESLWQSL  869
Consensus_aa:                 L..DsoA.GPl..@.pL@Ps..@h.sot.F.hR.Rsss.SshPYPs..DCLL.tlp.AT.ls.psLWphL
Consensus_ss:                                   eehhhh          eeeee          hh    hhhhhhhhh   hhhhhhhh

Conservation:                     6996 8     6    9959 97   97   5 8 6 6 6         95 66     9 6 8
YP_009505639.1_BlVS      769  VTNLPDCLLAPCEISKHGLSTDHFAVLAFFYSLRVTFLTSHGPVDLGMSDATTNFRIDHQPESKDL----  834
MZ422607.1_GaMV          780  CVNLPDSMLDADTIRARGLSTDHLVVLARFFNFRATLDLKPSPVTYGLSDSPVHFHITYTPESHTADGFK  849
ADD13602.1_OBDV          760  TATCPDSFLDPKSIAQHGLSTDHFVILAHRFSLCANFHSAAHVIQLGMADATSTFMINHTAGSAGL----  825
AYC35261.1_CSDaV         924  CAACPDSLLDPDIIRRVGLSTDHFAILAHHYSLRCRFHTAHGVIELGMADATSSFDIDHTAGN-------  986
QLM05437.1_GAMaV         882  CATCPDSMLRPEDIRREGLSTDHFAVLAHHYSLAADFHSASGIIPIGMADASAKFAITHTAGSGSA----  947
YP_009222597.1_NeVM      776  TSACPDSFLNPTEIAAAGLSTDHFAILARHYSLRATFHSGPSTFTIGMEDATSTFAINHTPGQGKL----  841
MZ305310.1_PGMV          745  TAELPDSMLNPIEVQTHGLSTNHFAVLARIYRLRARFLTKFGDITLGVQDATSSFTIRHTGEYAG-----  809
QID59334.1_SbMV          714  CSQLPDSQLQGDDISKGGLTTDHFVVLARIYRLRATFHTAAGEVTLGMENATSSFHIQHTAGN-------  776
QID59002.1_CaMaV         865  ITTLPDCLLDSSEIRVHGLSTDHFCVLALMHSLRARFISEGIDAELGLENAVHTFTINHFPGKDGS----  930
QQG34656.1_DiMV2         903  CNTCPDSLIDPKEIEVHGLSTDHFAILARAFSLKCTFITKDLRVDLGMHDATTSFSIDHTPGS-------  965
YP_004464920.1_SwMV      774  AANLPDSILDDGSLVRLGLTTDHFAVLARIFSLRCRFVSDHIDVELGVADATSRFTIRHT----------  833
NP_115454.2_MRFV         744  CANLPDSVLDDGSLATLGLTTDHFAVLARIFSLRCRFVSEHGDVELGLHDATSRFTIRHT----------  803
QCC30252.1_PeDV          782  CVQLPDSVLDYELIQRRGLSTLHFSVLARFFSLRAKFLTPGWEQPLGMTDATHTFTVLHTPPTATT----  847
SBuMV-BU1                850  CATLPDSFLSPSVIASHGLNTDHFCALAHNFSLQCHFHSGSNIQTMGLTNASHVFHITHDPPTPLQ----  915
YP_003475889.1_OLV-3     896  CSNLPDSFLDPQLIAKHGLSTDHFAVLARLFSLRCTFQSRSGTQTMGVADATHTFRIRHTPPTEDA----  961
YP_009551972.1_AVF       819  CTHLPDHFLDHNDIVRRGLNTKHLTTLCFVYKLSCTIHTEGRSHLFGLKNSTNHFNLDHNPEG-------  881
ATJ00054.1_MsMV1         802  CTHLPDSFLQHEDVLRAGLNTKHLVVLCYVFKLSCTINTEGRSHLFGVKNSTNHFTLDHDPRG-------  864
QQG34652.1_DiMV1         952  TSHLPDSLLDTAEITQHGLNTDHFTVLASIYDLKCTFLTNHGPIEFGVANAPTSFTIRHTPPTSNA---- 1017
YP_002756536.1_GSyV-1    819  CSYFPDSMLREEDIAKHGLSTHHFAALAREHRLQATFHSAGNQFVLGVEHPSVSFHIDHTPESATA----  884
QUS93818.1_GRVFV         870  CGILPDSQLDNEQVRTQGLSTDHFCALAYLHSLRCTFLTDAGQQEMGMEDATSVFTIRHTSGN-------  932
Consensus_aa:                 hs.hPDShLp..pl.p.GLsTcHFhhLA.h@pLptpFho.....hGh.sAsppFpIpHhs.s.......
Consensus_ss:                 hhh  hhh  hhhh      hhhhhhhhhhh eeeeeee   eeeee    eeeee      hhh
```

PRO II   CS   HEL[1]

```
Conservation:                 5999 5                6 75     6 78      5 8        996    59 5 7 6 99999699899
YP_009505639.1_BlVS      835  MGHFSLH-----QDSKPVPTLNGGT--GSELATAATRFNL-DGCLLPFNTVHTFVTAPSRAKNLISNMKN  896
MZ422607.1_GaMV          850  PGHFALS-------TARPANLNGGL--APDLATAALRFNT-GGALLPFHTVHTYTTQPARAKNLISNMKN  909
ADD13602.1_OBDV          826  PGHFSLR------LGDQPRALNGGL--AQDLAVAALRFNI-SGDLLPTRSVHTYRSWPKRAKNLVSNMKN  886
AYC35261.1_CSDaV         987  PGHFSLR-------QSATPRLNGGI--AQDLAVAALRFNI-DGTLLPIRSVHVYSTWPKRAKNLSSNMKN 1046
QLM05437.1_GAMaV         948  PGHFALR-------LPDSPKLNGGL--AQDLAVAALRFNH-SGALLPFRTAHPYTTWPRRAKNLSSNMKN 1007
YP_009222597.1_NeVM      842  PGHFSLR------LDHNSPKLNGGL--AQDLAVAALRFNV-DGQLLPIRSVHSYRTWPARAKNLISNMKN  902
MZ305310.1_PGMV          810  MGHFELV---ADTETSLPPPINGGG--AEDLAAAIMRYHAPDGTVLPIFQVHTRVTKPPRAKNLVSNMKN  874
QID59334.1_SbMV          777  PGHFALIRPPTDLASTLIPKVNGG---ARDLESSIKAFRAKDGSLLPFLKVHQYTTDPSRAKNLISNMKN  843
QID59002.1_CaMaV         931  IGHFALK------TPAHASKLNGGL--ADDLAAAALRFRTKGGHFLPFREAHYYKSSPARAKNLISNMKN  992
QQG34656.1_DiMV2         966  PGHFEFC-------PNPALTLTGSL--ADDLAHAALRFNI-KGVLLPFNTVHHYHSKPSRAKNLISNMKN 1025
YP_004464920.1_SwMV      834  PGHFELV----ADDYSLPALVGASTIPGADLAESCKRFVASDRTVLPFRDVHIHRTSVKRAKNLISNMKN  899
NP_115454.2_MRFV         804  PGHFELV----ADNFSLPALVGASSVPGADLAEACKRFVAPDRTVLPFRDVHIHRTDVRRAKNLISNMKN  869
QCC30252.1_PeDV          848  IGHFELV---ADGTSAPPPALSGAG--AKDLAIAIALSFRT-GGHLLPIAHVHNYTTAPSRAKNLISNMKN  911
SBuMV-BU1                916  PGHFTYL-------PTGNSVLTGAF--AEDLAQIALMFRV-HDHLLPFRTVHTYRTHPIRAKNLVSNMKN  975
YP_003475889.1_OLV-3     962  PGHFEYM-------EEIPESLTGAF--AQDLAHFALSFRF-NGYLLPFKHVHTYTSNVSRAKNLISNMKN 1021
YP_009551972.1_AVF       882  IGHFSHS------TRPSHIQLNGAR--AQDLSHTILGFRCSDSTLLPFKNIHSYSTHVSRAKNLISNMKN  943
ATJ00054.1_MsMV1         865  IGHFTHS------TKVQPVTLNGAR--AQDLKHTILGFRCSDSTILPFKNIHSYTTHVHRAKNLISNMKN  926
QQG34652.1_DiMV1        1018  PGHFELV------SDAQPPKLNGSR--TADLQHIMKSFRS-DNALLPFQKFHTYMSNTRRAKNLISNLKN 1078
YP_002756536.1_GSyV-1    885  PGHFSLR----ADERQHSPRLLGGR--AADLVHAALKFKV-GSAVLPFQQAHDYTTNVARAKNLISNMKN  947
QUS93818.1_GRVFV         933  PGHFELV------RSSARPLLNGAR-PAADIVEHMKRFKV-DGKILPFCEAHSYTSHVGRAKNLISNMKN  994
Consensus_aa:                 .GHFph.........s.s..LsGt...t.DLs.hhb.Fph.sshlLPhpphH.Ypo.s.RAKNLlSNMKN
Consensus_ss:                    ee                          hhhhhhhhh                    eee hhhhhhhhhhhhh
```

```
                                     MarBox-like              HEL I               HEL Ia
Conservation:                  9999779 559  6  69  55 89  5866 67 6 9697899799997 97  99    8   75789
YP_009505639.1_BlVS      897   GFDGVLANIDPHHTSTARDRLLCLDGIIDVAKPRRVRLFHIAGFAGCGKSYPIARLLKTPLFREFKVAVP   966
MZ422607.1_GaMV          910   GFDGILASIDPAHPSEARARLLTLDGVMDVASPRSVRLIHLAGFAGCGKSYPVSRLLKTATFKDFKLSVP   979
ADD13602.1_OBDV          887   GFDGVMASINPIRPSDAREKIVALDGLLDIAQPRSVRLIHIAGFPGCGKTHPITKLLHTAAFRDFKLAVP   956
AYC35261.1_CSDaV        1047   GFDGIMANIHPTKTNESREKILALDSQLDIAVRRSVRLIHIAGFPGCGKSFPISRLLRTPTFRNFKVAVP  1116
QLM05437.1_GAMaV        1008   GFDGVMATINPAKPSDAREKIIALDSSLDIAAPRSVRLIHIAGFPGCGKSYPIARLLSTPAFRNFKVAVP  1077
YP_009222597.1_NeVM      903   GFDGVMANIHPTKTTEARERILALDGQLDIAQPRTVRLIHIAGFPGCGKSYPISRLLKTAAFKDHKVAVP   972
MZ305310.1_PGMV          875   GFDGIMANVDPAHPNFAREQILKLDGVLDISVPRKVRLVHIAGFAGCGKSWPIAKLLTTPSFRNHKIAVP   944
QID59334.1_SbMV          844   GFDGVLANIDPTHPNTARDRIVALDGVMDIAQPRSLKLVHIAGFAGCGKSWPIAGLLNSPAFHNHKVAVP   913
QID59002.1_CaMaV         993   GFDGVMANVDPGHAQEARTRILSLDGVMDISCPRNVRLVHLAGFAGCGKSWPIAQLLKTPNFRNFKVAVP  1062
QQG34656.1_DiMV2        1026   GFDGILANADPLHTNEARDRLLSLDGIIDISMPKHVRLVHIAGFAGCGKSYPVSKLLKSPSFKNFKLSVP  1095
YP_004464920.1_SwMV      900   GFDGVMAQANPLDPKSARERFLMLDSCLDIAAPRVVRLIHIAGFAGCGKSWPVAQLLKTPAFRTFKLAVP   969
NP_115454.2_MRFV         870   GFDGVMAQANPLDPKSARERFLMLDSCLDIAAPRRVRLIHIAGFAGCGKSWPISHLLRTPAFRVFKLAVP   939
QCC30252.1_PeDV          912   GFDGVLANIDPHRTHEARDRLLALDGIMDLAPPRQVALIHIAGFAGCGKSYPIQKLLSHPAFHQHKVALP   981
SBuMV-BU1                976   GFDGVMASADPQNPKLARERILSLDGVLDIATPRSVRLIHIAGFPGCGKTYPITRLLSHPSFRNFRVAVP  1045
YP_003475889.1_OLV-3    1022   GFDGVMASADPLHPGQTRERFLSMDNQLDIAVPRETRLIHISGFAGCGKTYPITQLLKKPVFRQFRVAVP  1091
YP_009551972.1_AVF       944   GFDGVMANVDPLHPSKARDSFLMLDQLLDVAASKSVSLVHLAGFAGCGKSYPVQQLLKTPAFQNFKVSVP  1013
ATJ00054.1_MsMV1         927   GFDGVMANVDPLHPNKARDSFLMLDQLLDVAAPKNVHLVHLAGFAGCGKSYPVQQLLRTPSFQNFKVSVP   996
QQG34652.1_DiMV1        1079   GFDGIMATVNPQDPNSARHFFMALDHQMDIAAPRQVHLVHLAGFAGCGKSYPIQQLLQHQSFRSSRVAVP  1148
YP_002756536.1_GSyV-1    948   GFDGVLANIDPAHTNESRDRLLSLDGAMDIAAPRDVKLIHIAGFAGCGKSYPIAQLLKSRAFKHFKIAVP  1017
QUS93818.1_GRVFV         995   GFDGVLANIDPQHTNEARDRLLSLDGSLDIAKPRTVSLIHIAGFAGCGKSYPICQLLKTKPCRNYKIAVP  1064
Consensus_aa:                  GFDGlhAshsP.+sppAR-phL.LDs.hDlt.PRpVpLlHlAGFsGCGKo@Pl.pLLppssF+p@KlAVP
Consensus_ss:                      eeee          hhhhhhhhh          eeeeeeee        hhhhhhhhhhh      eeeee
```

```
                                 HEL Ia                               HEL II                      HEL III
Conservation:                  77999 999  5      99 799998999 786898997997999969966785      668
YP_009505639.1_BlVS      967   TVELRAEWKDLLKIRPGAQWRLSTWESSLLKSARILVIDEVYKMPRGYVDLAVHADPT-----IELVIL-  1030
MZ422607.1_GaMV          980   TTELRNEWKGILNLPAGSQWRLSTWESSLLKSARILVIDEIYKMPRGYLDLCIHADPT-----VEFVIA-  1043
ADD13602.1_OBDV          957   TTELRSEWKELMKLSPSQAWRFGTWESSLLKSARILVIDEIYKLPRGYLDLAIHSDSS-----IEFVIA-  1020
AYC35261.1_CSDaV        1117   TVELRAEWKTITGLPASEAWRIGTWESSLLKSARVLVIDEIYKMPRGYIDLAIHSDPT-----IEMVIA-  1180
QLM05437.1_GAMaV        1078   TVELRAEWKDLLKLPGNAAWRIGTWESSLLKMARVLVIDEIYKMPRGYLDLAIHADPT-----IEMVIA-  1141
YP_009222597.1_NeVM      973   TTELRAEWKDMLKPSPAHVWRLGTWESSLLKAARVLVIDEIYKLPRGYLDLAIHADPT-----IEFVIA-  1036
MZ305310.1_PGMV          945   TVELRAEWKELMNYRANEAWRVGTWESSLLKSARVLVVDEVYKMPRGYVDLAIHSDAA-----VEFVIL-  1008
QID59334.1_SbMV          914   TVELRTEWKDLLQSKPMDRWRVGTWESSLLKSARVLVIDEIYKMPRGYLDLALHADSN-----VDFVIL-   977
QID59002.1_CaMaV        1063   TVELRTEWKELLSSPPSERWRVGTWESSLLKSARVLVIDEVYKMPRGYLDLAIHSDSS-----VEFVFA-  1126
QQG34656.1_DiMV2        1096   TVELRSEWKELLNLKKGQEWRVGTWESCLLKSARVLVIDEVYKMPRGYLDLSIHADPA-----LEFLIV-  1159
YP_004464920.1_SwMV      970   TTELRDEWKGLMEPRDQDKWRFGTWESSLLKSARTLVIDEVYKMPRGYLDLAIHADAS-----IQFVIL-  1033
NP_115454.2_MRFV         940   TTELRDEWKALMDPRDQDKWRFGTWESSLLKTARVLVIDEVYKMPRGYLDLAIHADAA-----IQFVIL-  1003
QCC30252.1_PeDV          982   TVELRNEWKAAMKPKPANVWRISTWESSLLKSARVLVIDEVYKLPRGYVDLAIHADPS-----VEFVIL-  1045
SBuMV-BU1               1046   TVELRSEWKDMLKLKPAERWRIGTWESSLLKSARVLVIDEIYKMPRGYLDLAIHSDSS-----IQFVIV-  1109
YP_003475889.1_OLV-3    1092   TTELRSEWKDLLTLEPADRWRIGTWESSLLKSSRVLVIDEVYKMPRGYLDL----RPTPLIRPIQFVIIP  1157
YP_009551972.1_AVF      1014   STELRSEWKEAIKTKNSDNWRISTWESSLLKTARVLVIDEIYKMPRGYLDLAIHADPT-----IEFVII-  1077
ATJ00054.1_MsMV1         997   STELRSEWKDAIKTKNSDNWRISTWESSLLKSARVLVIDEIYKMPRGYLDLAIHADPT-----VEFVII-  1060
QQG34652.1_DiMV1        1149   TTELRTEWKEGMHLKSTTAWRVSTWESSLLKRSRVLVIDEVYKMPRGYLDLSILADPT-----LELVIL-  1212
YP_002756536.1_GSyV-1   1018   TVELRNEWKGVLKVKPQDNWRISTWESSLLKSARILVIDEIYKMPRGYLDLAIHADPT-----IDLVIA-  1081
QUS93818.1_GRVFV        1065   TVELRNEWKDSLKSAPADRWRLGTWESSLLKSARIVVIDEVYKLPRGYLDLAIHADST-----AQLFIV-  1128
Consensus_aa:                  ThELRsEWK.hhp..ssp.WRltTWESSLLKoARlLVIDElYKMPRGYlDLAIHtDso.....lphVIh.
Consensus_ss:                    hhhhhhhhhh           eeeeehhh            eeeee          hhhhhhhhhh          eeee
```

```
                                HEL III                            HEL IV
Conservation:                  88896997988666 969 77 69   59 57 9 99 985959   66  7 6 7 7   95        6
YP_009505639.1_BlVS     1031   LGDPLQGVYHSTHPSSTNSRLSPETHYLSKYIDFYCLWSHRIPQDVAKFFGVHSTNLAPGFSKWVPNLSP  1100
MZ422607.1_GaMV         1044   LGDPLQGEYHSTSPSSSNSRLSSEVRHLAPFIDYYCMWSRRIPKLVADFFQLPTLSSEPGFTRLIRHLPS  1113
ADD13602.1_OBDV         1021   LGDPLQGEYHSTHPSSSNSRLIPEVSHLAPYLDYYCLWSYRVPQDVATFFQVQSHNPALGFARLSKQFPT  1090
AYC35261.1_CSDaV        1181   LGDPLQGEYHSTHPSSTNSRLLSEPQHLSMYLDFYCLWSHRVPQNVAAFFHVKTTSKQPGFCRYQRELP-  1249
QLM05437.1_GAMaV        1142   LGDPLQGEYHSTHASSTNARLLPEPRHLAPYLDYYCLWSHRIPRNVAAFFQVPTSSTEEGFVRHVRELPG  1211
YP_009222597.1_NeVM     1037   LGDPLQGEYHSTHPNSSNSRLVGETRHLAPYLDYYCLWSRRVPKLIADFFHVPTSNENPGFARYSRQFPT  1106
MZ305310.1_PGMV         1009   LGDPLQGEYHASHPQSTNARLHSEIIELSPYIDAYCWWSSRRIPKRIAKCLGVPTLSSKQGFVSFRRELPS 1078
QID59334.1_SbMV          978   LGDPLQGEYHSTHASSSNHRLSSEVHRLRPYIDVYCAWTRRLPRRIARIFNIPTTSEHEGFVTYSKGFPH  1047
QID59002.1_CaMaV        1127   LGDPLQGEYHSTHPSSSNPRLPSEISHLRRYIDFYCFWSHRIPRNVAQCLGVPTTSKLEGFATYTQHFPT  1196
QQG34656.1_DiMV2        1160   LGDPLQGEYHSHHPSSTNARLSSEILHLNPFIDFYCMWSHRIPQDLARFFQIKSSNTSPGFRHHHLNLPS  1229
YP_004464920.1_SwMV     1034   LGDPIQGEYHSTHPSSSNARLSPEHVYLRPYIDFYCFWSRRIPQYVATILGVPSTSKVSGYARHSSQFPL  1103
NP_115454.2_MRFV        1004   LGDPIQGEYHSTHPSSSNARLSPEHRYLRPYVDFYCFWSRRIPQNVARVLDVPTTSTEMGFARYSQQFPF  1073
QCC30252.1_PeDV         1046   LGDPLQGEYHSTHPDSSNGRLSSELKHLRPYLDFYCLWSRRIPQQIARFFQVPTLSDAPGYVRYMRSFST  1115
SBuMV-BU1               1110   LGDPLQGEYHSTSPDSSNSRLSPETSHLSRYIDFYCLWSHRVPHLISGVFQVPTSSSNPGFVSHHRSLPP  1179
YP_003475889.1_OLV-3    1158   WRSPSQGEYNSTHPHSSNLRITSEIIHLQPYIDFYCFWSYRIPKNVAACLKVPTTSNKTGFIRRLNSIPN  1227
YP_009551972.1_AVF      1078   LGDPLQGEYHSSNPSSSNHKLSPETSHLRKYIDFYCFWSRRIPQSVAAFFQVPTLNSTPGFSKFVPSVPT  1147
ATJ00054.1_MsMV1        1061   LGDPLQGEYHSSNPSSSNHRLSPETLHLKPYIDFYCFWSRRLPQSIAAFFQVPTLNSNPGFSKFVPSVPS  1130
QQG34652.1_DiMV1        1213   LGDPLQGEYHSTHPSSSNHKISSEIPHLKPYIDYYCLWSRRVPQVVAKFFNIPSLNPNLGSFSYTPHLPE  1282
YP_002756536.1_GSyV-1   1082   LGDPLQGVYHSTHSDSSNHRLSSEVKHLQPYMDYYCLWSHRVPQDIGTFFGIKSTSTVPGFKSYQANIPS  1151
QUS93818.1_GRVFV        1129   LGDPLQGEYHSTNPNSSNSRLASEIKHLQPYMDMYCMWSHRIPRNVARFFRVKTTSSVEGSVTTSLVLAP  1198
Consensus_aa:                  LGDPLQGEYHSopPsSoN.RLssEh.+L..YlDhYChWS+RlPp.lA.hh.l.o.sp..G@.p@..phss
Consensus_ss:                  e                 hhhhh     hhhhh       eeee        hhhhhhh            eeeee
```

```
                                                   HEL V                  HEL VI
Conservation:                    8 67      56 5596 7 877999979    6   5699676  99   68699 9995 66 8699
YP_009505639.1_BlVS      1101   SSKILTNSQNSMKTLVDCGFASVTIASSQGSTYPGATNILLDRNSALLSHSNSLVALTRSKKGVVFTGDR 1170
MZ422607.1_GaMV          1114   QAKILTNSQNSSRVLNDCGRASTTIASSQGSTYDGPSYILLDRHTALLSPAHSLVALTRSKVGIIFVGDV 1183
ADD13602.1_OBDV          1091   TGRVLTNSQNSMLTMTQCGYSAVTIASSQGSTYSGATHIHLDRNSSLLSPSNSLVALTRSRTGVFFSGDP 1160
AYC35261.1_CSDaV         1250   NSRILANSQNAGHTLQQCGYAAVTIASSQGSTYENAACIHLDRNSSLLSPAHSMVALTRSKVGVVFTGDP 1319
QLM05437.1_GAMaV         1212   DSRILTNSQNAGHTPQQCGYAAVTIASSQGSTYPGAACIHLDRNSSLLSHAHPLVALTRSRKGVLFTGDL 1281
YP_009222597.1_NeVM      1107   DARVLTNSQNAMHTMNQCGYQSVTIASSQGSTYSGAACIHLDKNSALLSHGHSLVALTRSRTGVIFTGDP 1176
MZ305310.1_PGMV          1079   KLRILVNSQSAMKTLNQLGYQAITIASSQGSTYRSPACIHLDRNSRLLSHQNSLVAVTRSTEGIIFTGDF 1148
QID59334.1_SbMV          1048   ARKVLANARTTATTLQQLGYDSVTIASSQGSTYDRPTVVHLDKNSRLLSHQHSLVAMTRSRRGIIFTGDA 1117
QID59002.1_CaMaV         1197   NTKILCNSMSACKTLQQCGYDSVTIASSQGSTYSGPACVHLDRNSRLLSASHSLVAITRSKAGVIFTGDR 1266
QQG34656.1_DiMV2         1230   TCKIMANSQNAARTLQDCGYSSVTIASSQGSTYSSASCIHLDRNSAVLSHSHSLVALTRSKSGIIFTGDK 1299
YP_004464920.1_SwMV      1104   NGKILISAMTSAKSLVDCGYQAITIASSQGSTVNGPVHIHLDNHARRLSHQHSLVAITRSRAGLVFTGDP 1173
NP_115454.2_MRFV         1074   FGKILISARDSAKSLADCGYHAVTIASSQGSTIAGPAYVHLDNHSRRLSHQHSLVAITRSKSGIVFTGDK 1143
QCC30252.1_PeDV          1116   SAKLLANSIPTAKTLQQVGYNAITISSSQGSTLDGPAFIHLDRHSTLLSHHHSLVALTRSRAGVVFTGDK 1185
SBuMV-BU1                1180   NSKILTCSQSSAKTLLQCGYSAVTIASSQGSTYEGPACIHLDRNSRSLSDSHSFVAVTRSKKGIIFTGDL 1249
YP_003475889.1_OLV-3     1228   ANKVLTCSQSSMKTLNQCGFSSVTIASSQGSTLQEAACIHLDRNSRNLSNSHSLVAITRSKSGIIFTGDF 1297
YP_009551972.1_AVF       1148   NTPLLVNSQSASQILQSAGFRALTIASSQGATFSAPTAIHLDKNTKSLSPQHSLVALTRSKSGILFTGDQ 1217
ATJ00054.1_MsMV1         1131   NTPLLVNSQSASQVLQAAGFRALTIASSQGATFSAPTAVHLDKNTKSLSPQHSLVALTRSKVGILFTGDQ 1200
QQG34652.1_DiMV1         1283   GYPVLACSNNQVQTLTQCGFRSVSVASSQGSTYTKGVVLHLDRNSKLLSLAHSLVALTRSKDLIIFSGDK 1352
YP_002756536.1_GSyV-1    1152   NLRQLANSQSAAKVLNQCGFSSVTIASSQGSTYSAPACIHLDRHSMSLSHAHSLVALTRSKSGVIFTGDK 1221
QUS93818.1_GRVFV         1199   GWKQLTNSMNAARTLNDCGFAATTIASSQGSTYNHPACINLDKNSSQLSHAHSLVALTRSKVGIMFTGDL 1268
Consensus_aa:                   ...lLhsSpst.poLpptG@.tlTIASSQGST@p.shhlhLD+pS..LS..hSLVAlTRS+.GllFTGD.
Consensus_ss:                      eeeeeehhhhhhhhhh        hhh          eeeeee           hhheeeeeee     eeeee   h

                                                                                CS  RdRp
Conservation:                     6       9   65           5   57         5  85  9   6 95
YP_009505639.1_BlVS      1171   KMLEEGPTSNLLFSRYFTGKVVSLAGLFPSELPKCPHLTVPLTSRSVKLSGA------------------ 1222
MZ422607.1_GaMV          1184   AQLHGGPTCNRLLSDFWAGRKVSIPGLFADVLPPSIFLSAPLTSRQRSVSGAGASSAPAWTPCSLRNGPE 1253
ADD13602.1_OBDV          1161   ALLNGGPNSNLMFSAFFQGKSRHIRDWFPTLFPTATLLLSPLRQRHNRLTGAL--APVEPSHLLLPDLPS 1228
AYC35261.1_CSDaV         1320   AQLSNAPSSNRMFSEFFSGRTRPLHDWFHNEFPKATILTEPLKTRGPRLTGA------------------ 1371
QLM05437.1_GAMaV         1282   GILTGHTTSNRMFAEFYSGKSRPLHDWFHAEFPTATIIHEPITKRHNNLKGA------------------ 1333
YP_009222597.1_NeVM      1177   SLLKGASTSNTMFSLLMSGKTRPIQDWFHREFPTCPVLKEPLKQRHNRLTGAH--GET------------ 1232
MZ305310.1_PGMV          1149   ALLDGTPSANYILSHVAQEKEVDLRALLSLIAPKVPYLVAPIKSRKLTLRGG------------------ 1200
QID59334.1_SbMV          1118   SILDGTSQANFIFSAMARNTPIDLKGVFGDLLPNCVSLSAPMKSRKVILSGA------------------ 1169
QID59002.1_CaMaV         1267   QLLDGTPSSNAMFSALFAGKPYSAAGQLSELFPPCPRISEPITSRRTLLKGAASHASLPQPSRPASHRIP 1336
QQG34656.1_DiMV2         1300   MLLSGTSTSNYLYSKYWNGDKVHLSSLFPSELPKCPHITEPITSRKLKLQGS------------------ 1351
YP_004464920.1_SwMV      1174   SVADGTSSSNLLFSAVLMNHPISVRSLFSALLPRCPFVESPPTSRSVLLRGA------------------ 1225
NP_115454.2_MRFV         1144   AAADGTSSANLLFSAVLLDRRLSVRSLFSALLPCCPFVTEPPTSRAVLLRGA------------------ 1195
QCC30252.1_PeDV          1186   SQLSGLSASNLMFSALASNKTVSLTALFPSEFYGLPTITEPLTNRRLTLTGG------------------ 1237
SBuMV-BU1                1250   SLLDGSPSSNRIFSAVFQKKSIPLRGLFSTLLPQCPLISAPLKSRNLLLSGAGERESSIFRVCSISHTNT 1319
YP_003475889.1_OLV-3     1298   HLLDGTTSSNYLFSCLAQGKSVDLEMLFPKTFPPCPRLLQPIRSRRTILVGSSEDWAQFYRPTPLEDFSR 1367
YP_009551972.1_AVF       1218   SLLTPSSTGNNMFIYFSRSIPINIFHLFHKLISGLHIIRAPLTSRNTVLHGG------------------ 1269
ATJ00054.1_MsMV1         1201   SLLTPSATGNNMFIYFSRGIPINIFHLFHKLITGLEIIRQPLTSRRTILSGG------------------ 1252
QQG34652.1_DiMV1         1353   HQL--LPGGNRLFTEFFSQRPVDLNHLFHNELSNIPLLHSPLTTRRPKLTGSSQIKANLAIHLTHHNLSS 1420
YP_002756536.1_GSyV-1    1222   -RVLEAPGGNLLFSSYFQEKKVDLRALFPTEFPCCPILLEPLKRRPTDLTGG------------------ 1272
QUS93818.1_GRVFV         1269   SRLNPGSSSNLLFSKFKQGESVDLRALFPFEFPCCPTISEPLSTRPKFCLGG------------------ 1320
Consensus_aa:                   ..LpsssstN.hFS.hhp.p.lsl..lF..bhs.hshlp.PlpsRp..LpGt..................
Consensus_ss:                   hhhhh     hhhhhhhh       hhhhhhhhh

Conservation:                                                                         6      5
YP_009505639.1_BlVS      1223   ----------------------------------------------GLYDHAIP-----FRSAS 1235
MZ422607.1_GaMV          1254   SPPP------------------------------------------SPYGCRLP-----IRGPN 1270
ADD13602.1_OBDV          1229   LLPL-------------------------------------PASGPYSRAFP-----VRSRF 1248
AYC35261.1_CSDaV         1372   --------------------------------------------ASPYSKAVP-----IRQAS 1385
QLM05437.1_GAMaV         1334   --------------------------------------------TNAYARAIP-----IRQPA 1347
YP_009222597.1_NeVM      1233   -------------------------------------------LTDPYCLALP-----IRLSS 1247
MZ305310.1_PGMV          1201   ------------------------------------------------VAGLP---------- 1205
QID59334.1_SbMV          1170   ---------------------------------------------------AP-----IRARD 1176
QID59002.1_CaMaV         1337   SRPH---------------------------------------FEEDPYSLALP-----IRDRF 1356
QQG34656.1_DiMV2         1352   ----------------------------------------------SCYSLAVP-----IRRPG 1364
YP_004464920.1_SwMV      1226   ----------------------------------------------GYGSAPP-----LRPRE 1237
NP_115454.2_MRFV         1196   ----------------------------------------------GYGVARP-----LRARD 1207
QCC30252.1_PeDV          1238   ----------------------------------------------DPYQLRLP-----IRGPS 1250
SBuMV-BU1                1320   DPPTLQFLESLPPCPLAPIPSSSTEESKASHAPMDRIPFPIDILSQEEFGEDDPYSKGFG-----IRAHT 1384
YP_003475889.1_OLV-3     1368   FQATPVPETAIPCPNGYQGSPEMI----EGPSHANLPFEADLDSIDPLEGETSPYSLGFG-----VHART 1428
YP_009551972.1_AVF       1270   ---------------------------------------------------AIP-----SSRLS 1277
ATJ00054.1_MsMV1         1253   ---------------------------------------------------ALP-----SSRLF 1260
QQG34652.1_DiMV1         1421   LLKPDTAP-------------------------------PSNVFHSNPGHVILPDPHNATAKSR 1453
YP_002756536.1_GSyV-1    1273   -------------------------------------------------APFP-----FRDEA 1281
QUS93818.1_GRVFV         1321   ----------------------------------------------APWLAAEH-----LSERA 1333
Consensus_aa:                   .........................................................shP.....hp...
Consensus_ss:
```

```
Conservation:                     5    95         6 6    6677669998597999 8 795             6    78
YP_009505639.1_BlVS   1236   APMIKSSVTSDVILDPVRPFLGDGDLNAPQISTHFLPETRRPLHFDIPSGKPSS-DSPASAEPTVPVHEP 1304
MZ422607.1_GaMV       1271   DPSVKSTEQRDV-ISHAPIVLGDGSLNAPQVSTHFLPETRRPLHFDLPSAVCSS-VSLSPAAPTATAIEP 1338
ADD13602.1_OBDV       1249   AAAVKPFDRSDV-LSWAPIAVGDGETNAPRIDTSFLPETRRPLHFDLPSFRPQAPPPPSDPAPSGTAFEP 1317
AYC35261.1_CSDaV      1386   TPALKPDFQGDV-IISAPIVLGSGELNAPQVSTHFLPETRRPLHWDIPSAIPESATRPDSTEPTTCHPEP 1454
QLM05437.1_GAMaV      1348   QPALKPSSTADV-VLSAEIAVGDGSYNQPQVSTHFLPETRRPLHFDLPSARAEPADLPQSTEPSTTAFEP 1416
YP_009222597.1_NeVM   1248   SPCIKPTEVSDI-ISADTVVLGDGALNRPQVSTHFLPEARRPLHSDLPSAVPSSELSPSSADFTTPVHEP 1316
MZ305310.1_PGMV       1206   -EKISPDYRGDVIIDCDAAFLGLGDECMPRVSTHFLPETRRPLHIDIASALPSSADRPQAPDHSATAYEP 1274
QID59334.1_SbMV       1177   APPLKPDYQGDVILDLSAPFLGLGEANTPQVSTHFLPETRRPLHLDIPSAKPENADRPTGPDHSDTAIEP 1246
QID59002.1_CaMaV      1357   SSAITPDFRGDVVIDHSHPVSGDGSANSPQISTHFLPETRRPLHLDIASAIPSSADRPSSTDPTDTAFEP 1426
QQG34656.1_DiMV2      1365   DPSVNSSFTADVCINQSSIISGTGELNAPQISTHFLPETRRPLHFDIPSAVPTA-CSLSDTDITTAYIEP 1433
YP_004464920.1_SwMV   1238   SPPMRPDFQGDVIADLSAPFLGDGSSNAPQVSTHFLPESRRPLHFDVPSARHQVADHPLKPDHSACAIEP 1307
NP_115454.2_MRFV      1208   APPLGPDYVGDVILDSSAPILGDGSANAPQVSTHFLPETRRPLHFDIPSARHQVADHPLAPDHSACAIEP 1277
QCC30252.1_PeDV       1251   DHGPSPTTRDDVFLTQEDIVSGDGELALQRVDTTFLPETRRPLHQDLPSAINEP-APPTSVKFSDSAYEP 1319
SBuMV-BU1             1385   ACVTDASSVADV-VAFAPLLHDFQPPVLPQVSTHFLPETRRPLHQDVPSALPEP-VSVSPVTHSDTAHEP 1452
YP_003475889.1_OLV-3  1429   ARTTNPEATGDI-ITQSEIVLGDGELNMPQVSTHFLPETRRPLHFDNPSALPSS-TVPSPVDLSRTAFEP 1496
YP_009551972.1_AVF    1278   SNPINSSFIGDA-FSTASIFSGDGLELNPRVSPHFLPETRLPLHFDLPSAQISSAQPSDEVACSATPFTP 1346
ATJ00054.1_MsMV1      1261   KPPINSSFVGDA-YSTATIFSGDGLEIAPRVSPHFLPETRLPLHFDIPSAQVSSANPSNETNPSATPFTP 1329
QQG34652.1_DiMV1      1454   YTSIDSNYTADIYLNQSNTYHTPTE-DTNQISTHFLPETRRPLHFDLPSTQTSDPNPSSAFLPSSSAPEP 1522
YP_002756536.1_GSyV-1 1282   -RVFNPERRDDV-FVEAAVVCGDGSSNAPQVSTHFLPETRRPLHFDLPSAKPEFAAHEAPAPLTDTFIEP 1349
QUS93818.1_GRVFV      1334   ---LGPTDLPDLLLPEAPIIRGNGESNAPQVSTHFLPETRRPLHFDVASAIPEPAKPSGVDPLDLASITP 1400
Consensus_aa:                s.shpss..sDl.ls.s..h.GsG..shPplSTHFLPETRRPLHbDlsSA..ps.s.s.ss..oshhhpP
Consensus_ss:                           ee                                                      ee

Conservation:                79989 97 66559 8 5875 698      95999   7 56 65576558 97 9 55 999995999
YP_009505639.1_BlVS   1305   VYPGETFENLAAHFLPCHDPEDREIHFRGQLSNQFP-HINRPFELACQSSSLLAAVHSERDDPTLLPASI 1373
MZ422607.1_GaMV       1339   VYPGETFENLLAHFLPPHSPEDREITYRSQLSNQFP-HINRPFELAAQPSSLLAAIHNEKNDPTLLAASI 1407
ADD13602.1_OBDV       1318   VYPGETFENLVAHFLPAHDPTDREIHWRGQLSNQFP-HIDKEYHLAAQPMTLLAPIHDSKHDPTLLAASI 1386
AYC35261.1_CSDaV      1455   VYPGETFENLAAHFLPAHDPTDREIYWQGQLSNQFP-HMDKEFHLAAQPMSLLAAVHQEKQDPTLLPASI 1523
QLM05437.1_GAMaV      1417   VYPGETFENLAAHFLPAHDPTDREIHWAGQLSNQFP-HINKEYHLAAQPLSLLAAIHSEKDDPTLLPASI 1485
YP_009222597.1_NeVM   1317   VYPGETFENLAAHFLPAHDPETREIVFRNTMSNQFP-HLNKDFHLSAQPSSLIAAIHSEKDDPTLLPASI 1385
MZ305310.1_PGMV       1275   IYPGETFENVAAHFLPAHDPADKEINFRGMLSNQFP-HINMPFELSAQPSNLLAAIHSSKHDPTLLPASI 1343
QID59334.1_SbMV       1247   IYPGESFEALAAHFLPAHDPETREIYYRGEMSSQFP-HLNRPFELSAQPSNLLAAIHAPKQDPTLLAASI 1315
QID59002.1_CaMaV      1427   VYPGETFENLAAHFLPAHDPEDKEIYFKGQLSSQFP-HLNRDWSLSCQPSSLLAAIHSSKQDPTLLPASI 1495
QQG34656.1_DiMV2      1434   VYPGETFENLAGHFLPCHDPAQREIHYMGQLSNQFP-HIDRPFHLSAQPSSLLAAIHDSKQDPTLLPASI 1502
YP_004464920.1_SwMV   1308   VYPGESFEQLAALFLPPTDAESKEIRFRGELSNQFP-HLDKPFEISAQPSSLLAPVHNSKHDPTLLPASI 1376
NP_115454.2_MRFV      1278   VYPGESFESLASLFLPPTDAESKETYFRGEMSNQFP-HLDKPFELGAQTSSLLAPLHNSKHDPTLLPASI 1346
QCC30252.1_PeDV       1320   VYPGEDFFALAAHFQPANDPETREILFRDTLSCQFP-FLDEPFEISCQPMALLAARHREKKDPTLLPASI 1388
SBuMV-BU1             1453   VYPSENFENLVAHFLPPRDPALREIVYQDQTSNQFP-LLDQPFHLSTLPSSLAAAIHSSKQDPTLLPASI 1521
YP_003475889.1_OLV-3  1497   VYPGETFENIAGHFMGPRDPEVLEIIHNDQMSNQFP-LLDQPFSLAAQPSSLMAAIHNSQNDPTLLPASI 1565
YP_009551972.1_AVF    1347   VYPGEDFFSLAAHFNPNHDPELKEILYRDACSNQFP-WVNLPFEISCQPSSLLAAIHSSKNDPTLLPASI 1415
ATJ00054.1_MsMV1      1330   IYPGEDFFSLAAHFNPCHDPELKEILYRDACSNQFP-WVNQPFEISCQPSSLLAAIHSSKNDPTLLPASI 1398
QQG34652.1_DiMV1      1523   VYPGENFESVASSFIPPTDPETREIRFHGELSNQFP-YVNMPFKLSSLPSSTLAAIHSSKHDPTLLPASI 1591
YP_002756536.1_GSyV-1 1350   VYPGETFENIAAHFLPAHDPEVKEILFKDQRSNQFP-FIDQPFHVGAQPASLCAAVHHSKKDPTLLAASI 1418
QUS93818.1_GRVFV      1401   VYPGESFENLARCFLPAHDPEQKEIWYRNQLSNQFP-HMDKEFHLSAQPSSLLAPVHSTARDPTLLKASI 1469
Consensus_aa:                VYPGEsFEslAthFlPs+DPp.+EIh@psphSsQFP.@lspsFclttQPtoLlAAlHspKpDPTLLsASI
Consensus_ss:                      hhhhhhhh         eeee                                   hhhhhhhh

Conservation:                 99999965  69 76 697 99  97 5 8587 9 6    5977 875999 6997799789885697
YP_009505639.1_BlVS   1374   PKRLRFRPSSAPYRLTAKDEVLGSLLYEGLCRAYHRNSFTVLPFNETLYLECIALNEFAQLSSKTQSVIM 1443
MZ422607.1_GaMV       1408   QKRLRFRPSDAPYQLTSKDEILGSLLYEAWCEAYHRSPSDTVPFDPVLFAECIALNEYAQLSSKTQSVII 1477
ADD13602.1_OBDV       1387   QKRLRFRPSASPYRITPRDELLGQLLYESLCRAYHRSPTSTHPFDEALFVECIDLNEFAQLTSKTQAVIM 1456
AYC35261.1_CSDaV      1524   QKRLRFRPSDRPYQITPKDEILGQLLFEGLCRAYHRSPFHTEAFDPVLFAECINLNEFAQLSSKTQATIM 1593
QLM05437.1_GAMaV      1486   QKRLRFRPTPAPYFISPRDEILGQLLFEGLCRGYHRSPLSSEPFDPVLYAECICLNEFAQLTSKTQAVIM 1555
YP_009222597.1_NeVM   1386   GKRLRFRPSDAPYQITSKDEILGQLLYEGWCRAYSRNPNAEEPFDEALFAECINLNEYAQLTSKTQAVIM 1455
MZ305310.1_PGMV       1344   SKRLRFRPSTRPYQITAKDELLGGLLFEGLCRAYHRSPELTHPFNEQLFIECIGLNEFAQLSSKTQAVIM 1413
QID59334.1_SbMV       1316   TKRLRFRPTSRPYEITSKDELLGSLLFESLCRAYRRHPHTSVPFNETLFIECINLNEFAQLTSKTQAVIM 1385
QID59002.1_CaMaV      1496   AKRLRFRPSSKPYQITAKDQVLGQLLFEGLCRAYRRNPSSTETFDETLFIECINLNEFAQLTSKTQSVIM 1565
QQG34656.1_DiMV2      1503   AKRLRFRPTSRPYTITARDELLGQLLFDSLCTAYHRSSSHLEDFNEQLFIECIALNEFAQLSSKTQNVIM 1572
YP_004464920.1_SwMV   1377   AKRLRFRHNDSAYTISARDEILGGLLYEAWCRAYRRSPLDIEPFDPVLYAECINLNEFAQLSSKTQATIM 1446
NP_115454.2_MRFV      1347   GKRLRFRHSEAPYVIAPRDEILGSLLYAAACRAYHRSPRDVEPFDPDLYAECINLNEFAQLSSKTQATIM 1416
QCC30252.1_PeDV       1389   PKRLRFRPSEAPYSITPKDEILGGILFNSLCRAYRRNPNDTVPFDEVLFAECIALNEFAQLTSKTQATIM 1458
SBuMV-BU1             1522   GKRLRFRPSNNPYHLTAKDEFLGGQLFEGLCRAYHRNPNDSHPFDPILFAECINLNEYAQLTSKSQSTII 1591
YP_003475889.1_OLV-3  1566   GKRLRFRPSNAPYPITAEDQILGSLLFEALCRAYRRHPEAVVPFNPIAFAECINLNEYAQLTSKTQAVII 1635
YP_009551972.1_AVF    1416   QKRLRFRPNSSPYQISTKDEVLGLILFNSLCRAYHRNPSDVVPFDELLFIECINANEFSQLSSKTQAVIM 1485
ATJ00054.1_MsMV1      1399   QKRLRFRPNSSPYSISTKDEVLGLILYNALCRAYNRNPSTVIPFDELLFIECINANEFAQLSSKTQAVIM 1468
QQG34652.1_DiMV1      1592   PKRLRFRHNDSPYQISPKDELLGTFLYTSLLKAYRRPLYSTEPFDEALFIECINANEFSQLTSKTQNVIM 1661
YP_002756536.1_GSyV-1 1419   EKRLRFRASDAPYQITAKDEILGSMLFEAHCRAMRRDPNVRVPFDEALFAECIALNEFAQLTSKTQAVIM 1488
QUS93818.1_GRVFV      1470   GKRLRFRPSDSPYRLTSKDEILGSLLFDAHCTAMLRSPCASVPFDENLFAECIALNEFAQLSSKTQAIIM 1539
Consensus_aa:                .KRLRFRsos.PYplos+DElLG.hL@pthC+AYpRsP.ss.PFs.hLFhECIslNEFAQLoSKTQthIh
Consensus_ss:                hhh     hhhhhhhhhh  hhhhhhhhhhhhh            hhhhhhhh hhhhhhhh   hhhhh
```

```
                                                    RdRp I                  RdRp II
Conservation:                  695 9999999678979979599969659698 99999999999987795899999998 69  769 56
YP_009505639.1_BlVS    1444  ANSKRSDPDWRYSAVRIFSKAQHKVNENSIFGNWKACQTLALMHDAVVLLLGPVKKYQRMFDAQDRPAHL 1513
MZ422607.1_GaMV        1478  ANASRSDPDWRWSVVRIFSKTQHKVNENSIFGSWKACQTLALMHDAVVLILGPVKKYQRIFDAQDRPSTL 1547
ADD13602.1_OBDV        1457  GNARRSDPDWRWSAVRIFSKTQHKVNEGSIFGAWKACQTLALMHDAVVLLLGPVKKYQRVFDARDRPAHL 1526
AYC35261.1_CSDaV       1594  GNARRSDPDWRWSAVRIFSKTQHKVNEGSIFGSWKACQTLALMHDAVVLILGPVKKYQRVFDQRDRPRHL 1663
QLM05437.1_GAMaV       1556  GNARRSDPDWRWSAVRIFSKAQHKVNEGSIFGPWKACQTLALMHDAVVLLLGPVKKYQRVFDTRDRPSTL 1625
YP_009222597.1_NeVM    1456  GNARRSDPDWRWSAVRIFSKAQHKVNEGSIFGSWKACQTLALMHDAVVLILGPVKKYQRMFDQRDRPSHL 1525
MZ305310.1_PGMV        1414  ANACRSDPDWRWTAVRIFAKAQHKVNEGSIFGPWKACQTLALMHDAVVLLLGPVKKYQRVFDQAERPSHL 1483
QID59334.1_SbMV        1386  ANASRSDPDWRWTAVRIFAKAQHKVNEGSIFGSWKACQTLALMHDAVVLLLGPVKKYQRIFDEIDRPDHL 1455
QID59002.1_CaMaV       1566  ANASRSDPDWRWSAVRIFSKSQHKVNEGSIFGPWKACQTLALMHDAIILLLGPVKKYQRVLDQRDRPAHL 1635
QQG34656.1_DiMV2       1573  SNASRSDPDWRWSAVRIFSKTQHKVNENSIFGDWKACQTLALMHDAIILLLGPVKKYQRIFDQRDCPPHL 1642
YP_004464920.1_SwMV    1447  ANANRSDPDWRWSAVRIFAKTQHKVNEGSLFGSWKACQTLALMHDAVVLLLGPVKKYQRVFDQRDRPSHL 1516
NP_115454.2_MRFV       1417  ANANRSDPDWRWSAVRIFAKTQHKVNEGSLFGSWKACQTLALMHDAVVLLLGPVKKYQRFFDQRDRPSTL 1486
QCC30252.1_PeDV        1459  ANANRSDPDWRYTAVRIFAKTQHKVNDGSIFGSWKACQTLALMHDAVVLILGPVKKYQRVFDQADRPSHI 1528
SBuMV-BU1              1592  ANARRSDPDWRYTAVRIFSKTQHKVNEGSLFADWKACQTLALMHDAVILLLGPVKKYQRHFDARDRPATI 1661
YP_003475889.1_OLV-3   1636  SNARRSDPDWRYSAVRIFSKTQHKVNDGSFFGSWKACQTLALMHDAVILLLGPVKKYQRLFDSEDRPSHI 1705
YP_009551972.1_AVF     1486  ANSDRSDPDWRWSAVKIFSKAQHKVNEGSLFGDWKACQTLALMHDAVILALGPVKKYQRLFDKQDQPSNI 1555
ATJ00054.1_MsMV1       1469  ANADRSDPDWRWSAVRIFSKAQHKVNEGSLFGDWKACQTLALMHDAVILALGPVKKYQRIFDKTDRPDNI 1538
QQG34652.1_DiMV1       1662  ANAYRSDPDWRWSAVRIFAKTQHKTNDASIFGNWKACQTLALMHDAVILLLGPVKKYQRIFDKRDRPSNI 1731
YP_002756536.1_GSyV-1  1489  ANHERSDPDWRYTAVRIFAKNQHKVNSGSLFGPWKACQTLALMHDAVILLFGPVKKYQLIHDERDRPEHI 1558
QUS93818.1_GRVFV       1540  ANRDRSDPDWRWSAVRIFAKTQHKVNDHSIFGGWKACQTLALMHDAVVLLLGPVKKYQRIFDAADRPSNI 1609
Consensus_aa:                tNtpRSDPDWR@oAVRIFtKhQHKVN-tSlFGsWKACQTLALMHDAVlLlLGPVKKYQRlFD.pDRPspl
Consensus_ss:                h          hhhhhhhhh                  eeeee hhhhhhhhhhhhhhhhhhhhh    e

                                    RdRp III            RdRp VIperm            RdRp IV
Conservation:                75797 89   76 986 695                         7999879899999998979  99
YP_009505639.1_BlVS    1514  YVHAGNTPAQMSSWCQNHLTDTVH---------------------LANDYTAFVQSQHGEAVVLERKKME 1562
MZ422607.1_GaMV        1548  YVHAGNTPPQMSTWCQANLTSAIK---------------------LANDYTAFDQSQHGEAVVFERKKME 1596
ADD13602.1_OBDV        1527  YIHAGQTPSSMSLWCQTHLTPAVK---------------------LANDYTAFDQSQHGEAVVLERKKME 1575
AYC35261.1_CSDaV       1664  YIHAGNTPSQMSNWCQQHLTTAVK---------------------LANDYTAFDQSQHGEAVVLERKKME 1712
QLM05437.1_GAMaV       1626  YLHAGHTPSQMSAWCQRHLTSKVK---------------------LANDYTAFDQSQHGEAVVLERKKME 1674
YP_009222597.1_NeVM    1526  YIHAGHTPTEMSTWCKKNLTTAVK---------------------LANDYTSFDQSQHGEAVVFERKKME 1574
MZ305310.1_PGMV        1484  YVHAGHTPFEMSRWCQENLTRGEH---------------------LANDYTAFDQSQHGEAVVLERKKME 1532
QID59334.1_SbMV        1456  YIHAGHTPFQMSKWCQQHLSSREH---------------------LANDYTAFDQSQHGEAVVLERKKME 1504
QID59002.1_CaMaV       1636  YIHAGHTPFEMLKWCQAHLSSAVH---------------------LANDYTAFDQSQHGEAVVLEALKMR 1684
QQG34656.1_DiMV2       1643  YIHAGHTPQQMSKWCQQHLTDNIH---------------------LANDYTAFDQSQHGEAVVLERKKME 1691
YP_004464920.1_SwMV    1517  YVHAGHTPFEMAEWCQQHLTPAIK---------------------LANDYTAFDQSQHGEAVVFERFKMS 1565
NP_115454.2_MRFV       1487  YVHAGHTPFEMADWCRAHLTPAVK---------------------LANDYTAFDQSQHGEAVVFERYKMN 1535
QCC30252.1_PeDV        1529  YIHASHTPFEMSEWCQANLTDAEH---------------------LANDYTAFDQSQHGEAVVLERKKME 1577
SBuMV-BU1              1662  YIHAGHTPQDMSKWCQQHLSNSIH---------------------VANDYSSFDQSQHGEAVVLERLKMQ 1710
YP_003475889.1_OLV-3   1706  YIHAGHTPQDMSNWCQQNLTDSIH---------------------LTNDYTSFDQSQHGEAVVLEQMKMA 1754
YP_009551972.1_AVF     1556  YIHASHTPFDLSLWCQQHLTFSPH---------------------IANDYTAFDQSQHGEAVVLERKKME 1604
ATJ00054.1_MsMV1       1539  YVHASHTPFDLSSWCQKHLTFSEH---------------------IANDYTAFDQSQHGEAVVLERKKME 1587
QQG34652.1_DiMV1       1732  YIHASKTPFDLSHWCQQHLTHSIH---------------------IANDYTAFDQSQHGEAVVLEVKKMQ 1780
YP_002756536.1_GSyV-1  1559  FIYAGRTPQEMSEWCQKFLTPRSASSPVPVMVSGDDSLIGCHPHFVANDYTAFDQSQHGEAAVLERLKME 1628
QUS93818.1_GRVFV       1610  FVYAGHTPSEMADWCRANLRPGER---------------------IANDYTSFDQSQHGEAVVFERKKME 1658
Consensus_aa:                YlHAt+TP.pMSpWCQ.pLo.s.+.....................lANDYTAFDQSQHGEAVVhEpbKMp
Consensus_ss:                ee     hhhhhhhhhhh     e                     eeehhhhhh    hhhhhhhhhhhh

                                                               RdRp V               RdRp VI
Conservation:                96699  88 99  89668599999999999999999989979996977  69     6 7799999776
YP_009505639.1_BlVS    1563  RLSIPQALIDLHVFLKTNVETQFGPLTCMRLTGEPGTYDDNSDYNLAVINLEYSASHVPTMISGDDSLLD 1632
MZ422607.1_GaMV        1597  RLSIPQDLIDLHLHLKTHVSTQFGPLTCMRLTGEPGTYDDNTDYNLAVINLEYFAQGVPTMVSGDDSVLD 1666
ADD13602.1_OBDV        1576  RLSIPDHLISLHVYLKTHVETQFGPLTCMRLTGEPGTYDDNTDYNLAVINLEYAAAHVPTMVSGDDSLLD 1645
AYC35261.1_CSDaV       1713  RLSIPQALIDLHIHLKTHVSTQFGPLTCMRLTGEPGTYDDNSDYNLAVINCEYMAANTPTMVSGDDSLLD 1782
QLM05437.1_GAMaV       1675  RLNIPQQLIDLHVYLKTHVSTQFGPLTCMRLTGEPGTYDDNSDYNLAVINCEYFASSTPVMVSGDDSLLD 1744
YP_009222597.1_NeVM    1575  RLNIPQHLIDLHCFLKTNVSTQFGPLTCMRLTGEPGTYDDNSDYNLAVINLEYAASAVPTMVSGDDSLLD 1644
MZ305310.1_PGMV        1533  RLSIPQHLIDLHVYLKTNVETQFGPLTCMRLTGEPGTYDDNTDYNIAIIHLEYAVGNTPLMVSGDDSLLD 1602
QID59334.1_SbMV        1505  RLNIPKHLIDLHVHLKTNVETQFGPLTCMRLTGEPGTYDDNTDYNLAVIHLEYAVGSTPCMVSGDDSLLD 1574
QID59002.1_CaMaV       1685  RAGIPEHFIHLHCQLKTTVETQFGPLTCMRLTGEPGTYDDNSDYNLAVLHLEYCMGDTPVMISGDDSLLD 1754
QQG34656.1_DiMV2       1692  RLNIPKHLIDLHVYLKTNVETQFGPLTCMRLTGEPGTYDDNTDYNLAIINLEYYAHNVPTMVSGDDSLLN 1761
YP_004464920.1_SwMV    1566  RLSIPQELIDLHVYLKTNVSTQFGPLTCMRLTGEPGTYDDNTDYNIAVLHLEYAVGSTPLMVSGDDSLLD 1635
NP_115454.2_MRFV       1536  RLSIPAELVDLHVYLKTNVSTQFGPLTCMRLTGEPGTYDDNTDYNIAVLHLEYAVGSTPLMVSGDDSLLD 1605
QCC30252.1_PeDV        1578  RLSIPESLIDLHVHLKTNVSTQFGPLTCMRLTGEPGTYDDNTDYNMAVIYTEYAADSVPLMVSGDDSLLD 1647
SBuMV-BU1              1711  RLSIPTHLIDLHVHLKCNVSTQFGPLTCMRLTGEPGTYDDNSDYNLAVICLQYALGTTPVMISGDDSLID 1780
YP_003475889.1_OLV-3   1755  RLSIPQHLIDLHTHLKCNVSTQFGPLTCMRLTGEPGTYDDNSDYNLAVIHLQYAVGSTPCMVSGDDSLLD 1824
YP_009551972.1_AVF     1605  RLSIPQFLINLHIHLKTNVSTQFGPLTCMRLTGEPGTYDDNTDYNLAVLFSEYNISSQAVLVSGDDSLID 1674
ATJ00054.1_MsMV1       1588  RLSIPQFLIDLHIHLKTNVSTQFGPLTCMRLTGEPGTYDDNTDYNLAVLFSEYNISNQAVLVSGDDSLID 1657
QQG34652.1_DiMV1       1781  RLNIPQHLIDLHIHLKTHVSTQFGPLTCMRLTGEPGTYDDNTDYNLAVLFSEYAITDEAVLVSGDDSLID 1850
YP_002756536.1_GSyV-1  1629  RVNIPEWLIALHIMIKTHITTQFGPLTCMRLTGEPGTYFDNSDYNLAVIFLEYSMSGQWL---------- 1688
QUS93818.1_GRVFV       1659  RLNIPRHLIDLHVHLKTNVSTQFGPLTCMRLTGEPGTYDDNTDYNLAVLHLEYLVGSTPCMVSGDDSLLG 1728
Consensus_aa:                RLsIPp.LIsLHh@LKTpVpTQFGPLTCMRLTGEPGTYDDNoDYNLAVl..EYhhss.shMVSGDDSLlD
Consensus_ss:                h    hhhhhhhhh     eee    hhhhh       hhhhhhhhhhhhhh       eeee   hhh
```

```
                                              RdRp VII                      RdRp VIII
Conservation:               9  56 9  5  696979979   6 59997666  966959 799997558 89 86 695 795 9
YP_009505639.1_BlVS    1633 FEPPRRPEWSALEPLLALRFKKERDRYATFCGYYVGKAGAVRSPIALFAKLMIAVDDGSLGDKLISYLAE 1702
MZ422607.1_GaMV        1667 FEPPRRPTWPAISKLLALRFKKESGKYITFCGFYVGTAGIVRSPIALFAKLAIATDDASMDDKLASYLTE 1736
ADD13602.1_OBDV        1646 FEPPRRPEWVAIEPLLALRFKKERGLYATFCGYYASRVGCVRSPIALFAKLAIAVDDSSISDKLAAYLME 1715
AYC35261.1_CSDaV       1783 REPPTRPEWVILQPLLSLRFKKERGRYATFCGYYASHVGCVRSPVALFAKLAIAVDDGSISDKMASYLSE 1852
QLM05437.1_GAMaV       1745 CEPPRRPEWVVIQPMLALRFKKERSRYATFCGYYASHVGCVRSPVALFAKIAIAVDDGSIADKLASYLAE 1814
YP_009222597.1_NeVM    1645 YEPKVRPEWNAIKPLLALRFKKERGLYATFCGYYCARVGCVRSPIALFAKLMIAEDDGSISDKLASYLME 1714
MZ305310.1_PGMV        1603 REPPVRPDWSYIKDLLALRFKKEKGRYATFCGYYVGADGAVRSPVALFAKLMIAVDDSSISDKLAAYVTE 1672
QID59334.1_SbMV        1575 QEPPVRDEWPALRELLALRFKKERGRYATFCGYYVGPAGCVRSPVALFAKLMIAVDDASIRDKIVAYLTE 1644
QID59002.1_CaMaV       1755 SEPPSRPEWSVVEPLLALRFKKERGRYATFCGYYVGSAGAVRSPIALFAKLAIAVDDLSIEDKLTSYLTE 1824
QQG34656.1_DiMV2       1762 VEPMRRPAWPKIQEHLALRFKKEKDTCLTFCGYYVGSAGCIRSPVALFAKLMIAVDDGSISEKMASYLTE 1831
YP_004464920.1_SwMV    1636 SEPPVRDQWAAVEPMLALTFKKERGRYATFCGYYVGFTGAVRSPPALFAKLMIAVDDESIDDKLIAYLTE 1705
NP_115454.2_MRFV       1606 SEPPVRDQWSAIAPMLALTFKKERGRYATFCGYYVGFTGAVRSPPALFAKLMIAVDDGSISDKLIAYLTE 1675
QCC30252.1_PeDV        1648 FEPPTLPTWAAIRPLLALRFKKERSLYPTFCGFNVGRAGALRAPRTLFAKIAIAVDDGSIEDKLASYLTE 1717
SBuMV-BU1              1781 SLPPPRDNWPALSNLLALRFKTEFDRYSLFCGYFVGPAGAIRAPRALFAKLAIAVDDGSIRDKMESYLSE 1850
YP_003475889.1_OLV-3   1825 SKPPIRDEWTGIAPLLALRFKTELDRYSLFCGYFVGSSGAVRCPRALFAKIMIAVDDGSIPLKIASYLTE 1894
YP_009551972.1_AVF     1675 SVPPTNPSWSHIAPLLSLRFKKEIDRYSLFCGYYVSPAGAVRSPKALFAKLMIAVDDQSISDKIASYITE 1744
ATJ00054.1_MsMV1       1658 SIPPTNPSWALIKPLLSLRFKKEIDKYALFCGYYVSPAGAVRSPKALFAKLMIAYDDKSIEDKLASYITE 1727
QQG34652.1_DiMV1       1851 RVPPTRPTWPAIQQLLHLRFKKELSRYSLFCGYFVGPEGAVRCPIALFAKILQAIDDSTIPDKMASYLTE 1920
YP_002756536.1_GSyV-1  1689 ---SENPLWPAIKPLLALRFKKEKTRYGNFCGYYVGAAGAVRMPRALFAKIFIAVEDASIADKMASYATE 1755
QUS93818.1_GRVFV       1729 REPPRNPIWPAVKPLLALRFKKERTRYGEFCGYYVGCEGCIRSPIALFAKLMVCVDDGSIEEKMISYATE 1798
Consensus_aa:               .bPs.pspWshlp.LLALRFKKE.s+YshFCGY@Vt.sGtlRtP.ALFAKlhIAVDD.SI.DKhhtYloE
Consensus_ss:                          hhhhhhh    eeeee     eeeeeeee        hhhhhhhh          hhhhhhhhhhh


Conservation:               9 5999696 59   995  8 79995899899 86   9  7 79665            667 5 5  6
YP_009505639.1_BlVS    1703 FAVGHSLGDPFWTLLPLEAVLFQSACFDFFCRRAPPALKISLKLGEVSESIMARLGRGLNWASSAVYSML 1772
MZ422607.1_GaMV        1737 FVVGHSLGDALWSLLPVEAVPFQSACFDFFCRHAPPALKLALRIGEVPEATLALLANNTRWASGAVFSLL 1806
ADD13602.1_OBDV        1716 FAVGHSLGDSLWSALPLSAVPFQSACFDFFCRRAPRDLKLALHLGEVPETIIQRL-SHLSWLSHAVYSLL 1784
AYC35261.1_CSDaV       1853 FALGHSLGDHLWEALPLEAVPFQSACFDFFCRRAPRYLKLSLMLGEVPESIIARIGSSLKWASHAIYTTL 1922
QLM05437.1_GAMaV       1815 FALGHSLGDSLWQALPLSAVPFQSACFDFFCRRAPPELKLSLKLGEVPEDIIARVGSNLRWATHAIFTTL 1884
YP_009222597.1_NeVM    1715 FSIGHSLGDELWQALPLATVSYQSACFDFFCRRAPPELKLALKLGEVPEEVVARIG-HFKWATHATYSLL 1783
MZ305310.1_PGMV        1673 FAVGHSLGDQLWNVLPIECVRYQSACFDFFCRKAPVEMKLCFKIGEVPLPLLERAFQHIKWASHAVYALL 1742
QID59334.1_SbMV        1645 FSVGHSLGDALWTVLPVEVVKFQSACFDFFCRHAKPELKLCLKIGEVPEPVLQAAFQHIKWATHAVYALL 1714
QID59002.1_CaMaV       1825 FSVGHSLGDSMWNCLPLDAVPYQSACFDFFCRHAPAHLKLSLRLGEVDESIMRRAFQHIKWATHAVYALL 1894
QQG34656.1_DiMV2       1832 FSVGHSLGDSFWQLLPVETVQYQSACFDFFCRHAPRDLKIALHLGEVPQTILERLGSAVSWATHAVYSML 1901
YP_004464920.1_SwMV    1706 FTVGHSLGDAFWTILPVETVPYQSACFDFFCRRAPAQAKVMLRLGEAPESLLSMAFQGLKWASHSVYALM 1775
NP_115454.2_MRFV       1676 FTVGHSSGDAFWTILPVEAVPYQSACFDFFCRRAPAQAKVMLRLGEAPESLLSLAFEGLKWASHSVYALM 1745
QCC30252.1_PeDV        1718 FSVGHSLGDELWKLLPIESVPFQSACFDFFCRKASPAQKLVLKIGEIPLELLEAAFEDAPWASHAVWALL 1787
SBuMV-BU1              1851 FVVGHSIGDSFWTLLPLPQIPYQSACFDFFCRNAPPHLKVAFRIGEIPLSVLEVIFENIKWASHATYSML 1920
YP_003475889.1_OLV-3   1895 FSIGHSLGDEFWQLLPVEQVVFQSACFDFFCRNCPPAAKLLLRLGEAPQSILEATFGKLKWASNAVYSML 1964
YP_009551972.1_AVF     1745 FSVGHSLGQNMWLSLPLDQVPFQSANFDFFCRFAPRSLKVALNIGEVPSSIMELILPFLKHVTNPIWALL 1814
ATJ00054.1_MsMV1       1728 FSVGHSLGQDMWLSLPLEQVSFQSANFDFFCRFAPRSLKVALNIGEVPYNTLQLILPFLKHVTNPIWALL 1797
QQG34652.1_DiMV1       1921 FSIGHSIGQPMWNLLPIHQVMYQSALFDFFCRAAPPSWKVALRIGEVEDSLLELLSSSLKWVSNPVFA-I 1989
YP_002756536.1_GSyV-1  1756 FAIGHSLGDALWSLLPVEEVVYQSAVFDFLCRNAPRELKLLFKLGPVERSVVEAVQEFATWASYAFYRFL 1825
QUS93818.1_GRVFV       1799 FSIGHSLGDAFWNLLPIDAVPYQSAVFDFFCRHAPEDVKVMFKLGRVEVSLFSHLLSKLTWLSYSSYAYL 1868
Consensus_aa:               FtlGHSLGc.hWphLPlc.V.@QSAhFDFFCRpAP..hKlhhplGEls.sllp.h.p.lpWhopth@thL
Consensus_ss:               hhhhhh  hhhhhh  hhhhhhhhhhhhhhhh   hhhhhhhh      hhhhhhh   hhhhhhhhhh


                                                     MarBox   Minor CP →
Conservation:                  6  5       5     5  5  75 585
YP_009505639.1_BlVS    1773 SSAQRRALLTSSRQGRSLPDNPEVSKLQGELLQSFQ--------------------------------FV 1810
MZ422607.1_GaMV        1807 PAAIRYRLLHSKFLTRSLPESPDVTALQGELLHMSQ---------------------------------L 1843
ADD13602.1_OBDV        1785 PSRLRLAILHSSRQHRSLPEDPAVSSLQGELLHTFHA-------------------------------PM 1823
AYC35261.1_CSDaV       1923 SSAARVAILRSSRNSRSMPDDPDTTLLQGELLQHFQ---------------------------------- 1958
QLM05437.1_GAMaV       1885 SSAARWAILHSSRSTRSQPEDPDVTRYQGELLQHLQ--------------------------------SV 1922
YP_009222597.1_NeVM    1784 NSAARQILLHSSRNPRSLPEDPDTTKYQGELLQTLQL-------------------------------SA 1822
MZ305310.1_PGMV        1743 NFKHRRQLLHSSRQGRSLPDDPEVSKLQGELLQTFQS--------------------NIKLPLSGGL--- 1789
QID59334.1_SbMV        1715 SVNHRRQILHSGRLGRSMPEDPQVSQLQGELLQSFQS--------------------SIHLPLRGGCMSR 1764
QID59002.1_CaMaV       1895 SAQDRRQILNSSRQSRSMPEDPEVAQLQAELGYFHLSGGSDGPSNPAPHRTPCGPGQVIFPSAGSPSGS- 1963
QQG34656.1_DiMV2       1902 NSSQRKAILHAHRPSKTSYQNPEVSQLQSELLHSLQ--------------------------------LA 1939
YP_004464920.1_SwMV    1776 NSSHRRQLLHSSRRPRSLPEDPEVSQLQGELLHQFQ---------------------SLHLPLRGA--SM 1822
NP_115454.2_MRFV       1746 NSSHRRQLLHSSRRPRSLPEDPEVSQLQGELLHQFQ---------------------SLHLPLRGG--HM 1792
QCC30252.1_PeDV        1788 RGASKSKIIAA-RKTRPMPEASDVSQHQGKLPYTFQ--------------------------------GL 1824
SBuMV-BU1              1921 SSAARFAVVKF-RQSRSFPEDPEVSKLQGHLRDFFMSYFQRNSRHRARNSQPPPPPPRPIPPPTPTPPP- 1988
YP_003475889.1_OLV-3   1965 SATARHLLIKS-RLTRPPPESVEVSQLQGQLLASFQHMFSRASK------------------------KS- 2009
YP_009551972.1_AVF     1815 SSAQRISFLKLSKLYRQ-NRLPIHSQHEGELLPFSNSSSSSLDFAPLADSNNFYSNSDQVYHPHSRSLP- 1882
ATJ00054.1_MsMV1       1798 SSAQRISFLKLTKNYRQ-NHLPVSTLHEGELLPMSN-LSSTFDSSPISNIDNFFSNTDQIYHPHSRSLP- 1864
QQG34652.1_DiMV1       1990 SSP----------------------HEGELLPS---FDSLTHSPDLLTQQTI--------HFPHHLSGA 2025
YP_002756536.1_GSyV-1  1826 NSAQRKVLLTRSPQLHFPGDAPEVSQLQGELLQSFS---------------------------------M 1862
QUS93818.1_GRVFV       1869 SSLQRRALRGKAVRQHFPGDSKEVTELQGELLHTFS--------------------------------MS 1906
Consensus_aa:               sts.R..llp.pp..+..scsP-lophQGcL...hp............... ................
Consensus_ss:               hhhhhhhhhhhh
```

```
                                                                                       Major CP
                                                                    CS
Conservation:                          6           55 5          5
YP_009505639.1_BlVS   1811  PQPSM---------NNSLLL-------------PLFGG---------LSPSLAAHPMPGPNVT-------- 1842
MZ422607.1_GaMV       1844  NQSSM---------HPDTLL-------------PLHGGS---SSNILPTPAIPSLPITPA-SMANASL--- 1885
ADD13602.1_OBDV       1824  PSPPS-------------L-------------PLFGG---LSPDNILTPHEFRTALYE--SSAYPTPPNS 1862
AYC35261.1_CSDaV      1959  -VPFM---------QSDTLL-------------PLTGGS--SAP--ILTPEAFSTSLAFSMASD------- 1995
QLM05437.1_GAMaV      1923  PSSRM---------NPDTLL-------------PLKGGS--SAP--QLTPEAFSLALAQSMATPN------ 1961
YP_009222597.1_NeVM   1823  PLHSM---------NHSTLL-------------PLTGGSSASAP--SLTPLQFSEAVTQSMASN------- 1862
MZ305310.1_PGMV       1790  MENAL---------NCSTLTPD-------VPLPLSGA-LNSGY--DPAPLYQSAQMTTLA---------- 1830
QID59334.1_SbMV       1765  AEAHQ-----------LLL-------------PLSGA--------ISTPPGQTFMALDA----------- 1791
QID59002.1_CaMaV      1964  PHHTL---------DCSTFSNCHMSDSLLPELYLSGGALNTPP--ALSPSPSLLFMASTP---------- 2012
QQG34656.1_DiMV2      1940  DQPSM---------QPETLL-------------PLTGAS--NHPSQHFNIPQFSLPMSDAS---------- 1976
YP_004464920.1_SwMV   1823  PNPLA---------APFRLL-------------PLGSAS--LGPSYSVAPLQPPPASESPDSMAENA---- 1865
NP_115454.2_MRFV      1793  PNPLA---------ALFRL-------------LQQSSS--LGPTYAVAPIARAPQVLPP-SMADNA---- 1833
QCC30252.1_PeDV       1825  TTHPL-------------M-------------TLSGAA---------SPANLAFLGSSAVPDLEPM---S 1856
SBuMV-BU1             1989  QPPSTSQSKPALHSSPSTFL------------PLLPEPVLLDP----VPVESALASPITPLDSSSVFDLP 2042
YP_003475889.1_OLV-3  2010  KSPSQSYQPRQPATPTSVL--------------SPPSSPPLSPSTFAPPPEPPPPPPPPPPPPPSVQGIP 2065
YP_009551972.1_AVF    1883  SNPLF----------------------------LSGGSMALSAIEALAQLLPLIQGGRDLLASTSDVSSP 1924
ATJ00054.1_MsMV1      1865  QPPIF----------------------------LSGGSMALTALEALTQLLPLIQGGKNLLTSTQDVPEQ 1906
QQG34652.1_DiMV1      2026  SHPPH-------------L-------------PMEELLALLPALRELLSKNSSDAHPSSPQTVA------ 2063
YP_002756536.1_GSyV-1 1863  MQPT--------------L-------------PLTGG--------LLLPPAVDAPMSDD-SLA------- 1889
QUS93818.1_GRVFV      1907  SPPSK----------------------------LTGG--------LLLPTSPSMSNEAT----------- 1929
Consensus_aa:               ..s...............L.............PL.tts...ss...hss...s.....s...........
Consensus_ss:                              eee                  hhh              hh

                                                                                      CP I
Conservation:                              55 5    6 8  6 59 88      75              9996       6
YP_009505639.1_BlVS   1843  ---------SQVGPAPP-NDDRVDRQPSLP----LAPRVAEVSPLHA--HIDYPFQWEVGTYSGDKAAFV 1896
MZ422607.1_GaMV       1886  ---------TQVGPAPA-ADSRIDAQPSLP----RAPRLLESSVPV---YIDYPFQWQIASYTGSASAFI 1938
ADD13602.1_OBDV       1863  PTSMSGIHASQVGPPPA-SDDRTDRQPSLP----LAPRLVESSLAVP--YVDVPFQWAVASYAGDSAKFL 1925
AYC35261.1_CSDaV      1996  ---------AQAGPAPS-RDDRVDRQPRLP----AAPRVAEVGLNAP--SVDYPFQWVVASYDGSEAKNL 2049
QLM05437.1_GAMaV      1962  ---------VQAGPPPP-SDDRTDRQPPLP----RAPRLVEDASAIP--FVDYPFQWVVASYDGLTAKNL 2015
YP_009222597.1_NeVM   1863  ---------GQVGPPPD-RDDRVDRQPRLP----VAPRVAEVDLQAP--TVDYPFQWVISSYDGSAAKNL 1916
MZ305310.1_PGMV       1831  ---------TNAGPAPQ-TDDRVDRQPLLP----PAPLVVETAAP----HIDLPFQWLLGNAPGDKNYYV 1882
QID59334.1_SbMV       1792  ---------TKVGPAPD-RDDRIDRQPPLP----APPRVLETQAPV---HVDVPFQWVVGSYAGEKNVFV 1844
QID59002.1_CaMaV      2013  ---------SDVGPPPQ-SDDRRDHQPRLP----APSRVVEALAPI---YIDYPFQWKVGTYTGAADVFV 2065
QQG34656.1_DiMV2      1977  ---------TQVGRPSD-RDDRSDPQPSLP----LAPRLRETKAQ----EILYPFQWVVGEYDGSKVEHL 2028
YP_004464920.1_SwMV   1866  ---------TQVGPAPA-RDDRLDRQPAIP----APPRVVETAPTH---YIDLPFQWKITDFTGAAAYHD 1918
NP_115454.2_MRFV      1834  ---------TQVGPVPP-RDDRVDRQPPLP----DPPRVLETAPSH---FLDLPFQWKVTDFTGYAAYHG 1886
QCC30252.1_PeDV       1857  NAQT-----PNVGPRPD-SDVRVDRQPLLPAPNPGPPAPLPSKNP----DVTVPFQWVISDFDGADVVHS 1916
SBuMV-BU1             2043  LHLMANVQTGQDSSAPPHRDDRFDPQPGLP----LAPSLSQTSNQAP--FVDVPFQWLVASLSGDRDTQT 2106
YP_003475889.1_OLV-3  2066  LHFMANTTPPPSGPAPP-RDDRVDHQPSLP----LAPQIQETTSSVP--HVDIPFQWLSASLTGEKDVQQ 2128
YP_009551972.1_AVF    1925  SSAQ-----AEKSPAGSSPDARVLRAPPLP----SPARQLSTPVSTSSLSIDLPFQWNFFDLTGTETKSA 1985
ATJ00054.1_MsMV1      1907  TAAQ-----TPSSPAGSSPDARVLRAPPLP----SPARQLPTEVSASSLSIELPFQWDFFDLTGTETKSA 1967
QQG34652.1_DiMV1      2064  ---------PAILPGSS-SGMRLVSPPRLP----APVRSLTTPASLAQDSISLPFQFTYYTITGVETGST 2119
YP_002756536.1_GSyV-1 1890  ---------GRARSQRD-PDHRVDPQPSLP----LAPSVQETPGGP---AITVPFQWVALVVKSESTIF- 1941
QUS93818.1_GRVFV      1930  ---------VPVSGRPSRDDHRSDPQPVLP----LAPKVQESQAL----ALSIPFQWVALIIKSDSAAF- 1981
Consensus_aa:               .........s.stsssss.pDsRhD.pP.LP.....ssp..ps.s.h....lshPFQW.h.shsG..s...
Consensus_ss:                                   eee                            eee   eeeeee

                                CP II                                                        CP III
Conservation:               7   6 5  7  5  7858 7  58    8   75  955   796      65   7   6966  5 9
YP_009505639.1_BlVS   1897  SDDLSASKTLKTLTAGYRHAEILSAEVDFVPLAPSFSKPISVGAVWTIASISPASAHEQSYYGGRLLTLG 1966
MZ422607.1_GaMV       1939  TDDLSASPTLKKVMAGYRHAELLHVELEFSPLQPSFSKPVSLSAVWTISSCLPTEGTETSFYGGRVFTIG 2008
ADD13602.1_OBDV       1926  TDDLSGSSHLSRLTIGYRHAELISAELEFAPLAAAFSKPISVTAVWTIASIAPATTTELQYYGGRLLTLG 1995
AYC35261.1_CSDaV      2050  SDDLSGSATLTKVMANYRHAELTSVELEVCPLAAAFSKPISVSAVWTIASISPASASETSYYGGRLFTVG 2119
QLM05437.1_GAMaV      2016  TDVLSGSKTLSTITANYRHAELLSVELEFAPLAGSFSKPITLSAVWTVGSITPATTTETSYYGGRVITIG 2085
YP_009222597.1_NeVM   1917  TEDLAGSATLSKLTANYRHAELRSVELEVAPLAAAFSKPISISVVWTIASITPATTTETSYYGGRLITVG 1986
MZ305310.1_PGMV       1883  TDAMATSVNISKLMAGYRHATLTKLEVEVVPLAAAFAKPITTTLVWTISSLTPSTTNELAYYGGRQLTLG 1952
QID59334.1_SbMV       1845  SHTLAAAGRLVNLTSLYRHAQLLHAEVEFAPTWNAFSKPVSASVVWTVADITPATTKEQEYYGGRYLTLG 1914
QID59002.1_CaMaV      2066  TDDLSGSATLKTITNGYRHAELISVEAEFSPLAASFGKPITFSVVWTVADVAPATLTETAYYGGRIITIG 2135
QQG34656.1_DiMV2      2029  TEDISASSSLNKVTAGYRHAELVSLELEFSPLAPSFSKPVTFTVVWTIASITPAQTKETTYFGGRVLTIG 2098
YP_004464920.1_SwMV   1919  TGDLSASPVLTTLCAPYRHAELISVELSLAPCPPSFTKPIMFTVVWTPATLSPTDGKETDFYGGRQISIG 1988
NP_115454.2_MRFV      1887  TDDLSASAVLTTLCAPYRHAELLYVEISVAPCPPSFSKPIMFTVVWTPATLSPADGKETDYYGGRQITVG 1956
QCC30252.1_PeDV       1917  TLDLSASSTLKKVTAYFRYAAIQSVEALIIADAAAISKPIPVSLVWTINTLTPDSGKELDYFGGQRIVAG 1986
SBuMV-BU1             2107  TTTLASSATLAKLTSIYRYAEIRSVELEFVPTSLSFSKPISVSAAWTIASITPAAADEMDYYGGRFLTVG 2176
YP_003475889.1_OLV-3  2129  TVSLSASQTLSKLWALYRHAELRSVEAEFVPTASSFSKPISISCAWTIASITPASGDETDYFGGRFLTIG 2198
YP_009551972.1_AVF    1986  SISIAGSSHITELLPQYRYARLINLELVVFPMAISMKYPQTVDVVWCTADQTITDGKIMATYGSQRISVG 2055
ATJ00054.1_MsMV1      1968  SLSIAGSTHITSLLPQYRYARLLQLELVVFPMSISMKYPQTVDVVWSTADQTITEDKIMATYGSQRISVG 2037
QQG34652.1_DiMV1      2120  SLDVSGKDVILNLIKPFSHARLISLEAVVFPYSASLTYPQTFDAVWSTADSVIAGADIINVYGAQRVTFG 2189
YP_002756536.1_GSyV-1 1942  TVDPPRAKSLTQLIGPYRHARLLSLEAILMPTLNAFQNPVTVHMVWTVNTVQPASGEELFYPGGQALTVG 2011
QUS93818.1_GRVFV      1982  TVDLAASTTLKKLTDPFRSCEITHLEVVLMPTLNAFNNPVTLHCVWTVNSIQPASGDELLYYGGQAITAG 2051
Consensus_aa:               o.slttttsslpplhs.YR@AcLhplEh.hhPh.sthppPlohshVWohssh..hsspbh.h@GGp.lolG
Consensus_ss:               eeeee      hhhh     eeeeeeeeeee      eeeeeeee        eeeee    eee
```

```
                                 CP III
Conservation:                    997 65     58667  588 66  8 7 9697                       5    569 5 56
YP_009505639.1_BlVS     1967  GPVLMSSTTRIPLDVSRINPVIKSSVSYSDTPRISYTVYS-AA----GTANTALISVIIRGIVRLSG--- 2028
MZ422607.1_GaMV         2009  GPVLMSSTTVLPADLNRLNPVILSPVAFTDCPRYSYTIYKCD-----SSNTSNLVSVLIRGVVRLSG--- 2070
ADD13602.1_OBDV         1996  GPVLMGSVTRIPADLTRLNPVIKTAVGFTDCPRFTYSVYANS-----GSANTPLITVMVRGVIRLSG--- 2057
AYC35261.1_CSDaV        2120  GPVLMSSTTHLPADLTRLNPVLKGPVKYTDCPRFSYSVYSNG-----GTKGTNLCTIILRGVVRLSG--- 2181
QLM05437.1_GAMaV        2086  GPVLMNSTTAVPADLRRLNPIIKDQISYTDCPRFSYSVYANG-----GTAGTNLVTVLIRGVVRLRS--- 2147
YP_009222597.1_NeVM     1987  GPVLLSSTTRIPADLSRLNPILKSSVGYKDTPRLSYTVYANS-----GTAGTNLCSVIIRGILRLSA--- 2048
MZ305310.1_PGMV         1953  GPVLMGSTTTVPCDLTRINPHLKSSVTYNDTPRLTYTTYANS-----GAANAPLINFIVRGIIRLSG--- 2014
QID59334.1_SbMV         1915  GPVAMGSTTIVPADLARINPVIKSAVTYIDGPRLSYTIYANG-----GTANTNLVNVTIRGSIRLSS--- 1976
QID59002.1_CaMaV        2136  GPVLMSSTTSVPADLSRLNAVIKASVSYRDTPRLSYTACSCG-----GSANTNLAQVTLRGVVRLSG--- 2197
QQG34656.1_DiMV2        2099  GPVLMSSTTSIPADLSRLNPVIKSSVPFSDTPRFTFTSPGVSS----ATKGSNLVSIILRGTIRLSG--- 2161
YP_004464920.1_SwMV     1989  GPVMLSSTTAIPADLTRMNPVIKSSVAYRDTPRWTMSVPAVSG----GDTKVNLATGFIRGVIRVSA--- 2051
NP_115454.2_MRFV        1957  GPVMLSSTTAVPADLARMNPFIKSSVSYNDTPRWTMSVPAVTG----GDTKIPLATAFVRGIVRVSA--- 2019
QCC30252.1_PeDV         1987  GPVSLATRNVIPADLTRLTPIIKDRVSYSDTPRLTWTAQKVAG----GAKGTTLCSLVIVGTILLSA--- 2049
SBuMV-BU1               2177  GPVLLSSTTHIPCPLNSINPTIKTSVSYSDTPRFTFTARAVSG----GSANAALALVIIRGVVRLSG--- 2239
YP_003475889.1_OLV-3    2199  GPVLLSSTTHIPCDLRSINPTLRSSVEFRDTPRVTFTARAVAG----ATANTPLAHFIIRGVLRLSG--- 2261
YP_009551972.1_AVF      2056  GPLNMSSHSILPANLSSLNPVVKDSVTYNDTPKLCVRFYENSDCKALGITAPICASLFIRGNIQLSS--- 2122
ATJ00054.1_MsMV1        2038  GPLNMSSHSILPANLSQLNPVVKDSVTYNDTPRINVRFFENADCKALGISAPVCASLFIRGKIRLSS--- 2104
QQG34652.1_DiMV1        2190  GPVSSSVLITIPADLKTMNPILRDSVTYKDCPKLNLAFYTNHDNLALGTKTPNCGAVIIRGVIQGSS--- 2256
YP_002756536.1_GSyV-1   2012  GPVSMSALATVPADVSRLNPVIKGAVAFLDTPRLTGTTMKCA-----KSETSPMAYVVIRGTLALSG--- 2073
QUS93818.1_GRVFV        2052  GPVSMNALATVPADLSRINPRIKSSVGYLDTPRLTGTTMKCA-----TAQTLPLAYVMIRGMVSVSG--- 2113
Consensus_aa:                 GPV.htthh.lPADLsplNPhlKstVs@.DhPRhohsh..ss.....ts.ssshh.lhlRG.lplSt...
Consensus_ss:                         eeee        eee        eeeee                    eeeeeeeeeeee


Conservation:                     8 5
YP_009505639.1_BlVS     2029  ---PSGNTVA----- 2035
MZ422607.1_GaMV         2071  ---PSGAKLT----- 2077
ADD13602.1_OBDV         2058  ---PSGNTVTATT-- 2067
AYC35261.1_CSDaV        2182  ---PSGNLLA----- 2188
QLM05437.1_GAMaV        2148  ---PSGNLLA----- 2154
YP_009222597.1_NeVM     2049  ---PAGNILA----- 2055
MZ305310.1_PGMV         2015  ---PSGNAF------ 2020
QID59334.1_SbMV         1977  ---PSGGLLTSD--- 1985
QID59002.1_CaMaV        2198  ---PTGNKLA----- 2204
QQG34656.1_DiMV2        2162  ---PSGNHLA----- 2168
YP_004464920.1_SwMV     2052  ---PSGAAIKAAS-- 2061
NP_115454.2_MRFV        2020  ---PSGAATPSA--- 2028
QCC30252.1_PeDV         2050  ---PTGTSI------ 2055
SBuMV-BU1               2240  ---PSGGKFT----- 2246
YP_003475889.1_OLV-3    2262  ---PSGAKFA----- 2268
YP_009551972.1_AVF      2123  ---PTATPSK----- 2129
ATJ00054.1_MsMV1        2105  ---PSATPTK----- 2111
QQG34652.1_DiMV1        2257  ---VRPTPTK----- 2263
YP_002756536.1_GSyV-1   2074  ---PVGTRLSE---- 2081
QUS93818.1_GRVFV        2114  ---PMGIKL------ 2119
Consensus_aa:                 ...Psts.h......
Consensus_ss:
```

h - alpha-helix; e - beta-strand; ***bold and uppercase letters*** - conserved aa; *l* - aliphatic (I, V, L); @ - aromatic (Y, H, W, F); *h* - hydrophobic (W, F, Y, M, L, I, V, A, C, T, H); *o* - alcohol (S, T); *p* - polar residues (D, E, H, K, N, Q, R, S, T); *t* - tiny (A, G, C, S); *s* - small (A, G, C, S, V, N, D, T, P); *b* - bulky residues (E, F, I, K, L, M, Q, R, W, Y); **+** - positively charged (K, R, H); **-** - negatively charged (D, E); charged (D, E, K, R, H); **bold** letters - conserved aa motifs; CS - protease cleavage site; highlighted in teal - protease catalytic diad aa, C and H; highlighted in yellow - start of PRO domain expressed in *E. coli*; highlighted in turquoise – tested cleavage site in *E. coli* [1]; MTase-Gtase - methyltransferase-guanylyltransferase; highlighted in bright green - marked conservative aa for MTase-Gtase domain; PRO - protease; TMD – transmembrane domain; Hel - helicase, RdRp - RNA dependent RNA polymerase; CP - coat protein; bold and light blue - putative M for major coat protein; highlighted in grey - sea buckthorn marafivirus (SBuMV) isolate BU1buckthorn marafivirus (SBuMV) isolate BU1.

References

1. Patel, A.; McBride, J.A.M.; Mark, B.L. The endopeptidase of the maize-affecting marafivirus type member maize rayado fino virus doubles as a deubiquitinase. *The Journal of biological chemistry* **2021**, *297*, 100957.

Table S1. Primers used for SBuMV CPs and genome fragment amplification and sequencing.

Primer name	Primer sequence	Application	Producer
M13seq-F	5′ GCC AGG GTT TTC CCA GTC ACG A 3′	Forward Sequencing Primer	metabion
M13seq-R	5′ GAG CGG ATA ACA ATT TCA CAC AGG 3′	Reverse Sequencing Primer	metabion
pJET1.2-F	5' CGA CTC ACT ATA GGG AGA GCG GC 3'	Forward Sequencing Primer	Thermo Fisher Scientific
pJET1.2-R	5' AAG AAC ATC GAT TTT CCA TGG CAG 3'	Reverse Sequencing Primer	Thermo Fisher Scientific
MetPro-seq2-R	5′ AGG GTT CAG AAT CTG GGT TAG AAA TGG GAA C 3′	Sequencing and 5′ RACE	metabion
MarRdRp-seq2-F	5′ ACC TCT CAC GTG CAT GAG ACT AAC TGG CGA 3′	Sequencing and 3′ RACE	metabion
MarCP-2F	5′ GGT GGT CGT TTC CTC ACG GT 3′	3′ RACE	metabion
MarHel-seq3F	5' TTG TCA TTG TCC TCG GAG ACC CTC TTC AAG GA 3'	Sequencing	metabion
MarRdRp-seq2-R	5′ TCG CCA GTT AGT CTC ATG CAC GTG AGA GGT 3′	Reverse Sequencing Primer	metabion
MarRdRp-seq3R	5′ GGA GGG TCG ATC GCG AGC ATC AAA GTG ACG T 3′	Sequencing	metabion
Mar-RdRp-R	5′ TAA AAG AAG TCA CGC AAA TGA CCC TGT AAT TTT GAC A 3′	HEL-RdRp fragment amplification and sequencing of PCR product	metabion
Mar-Hel-F	5′ GTT CTT GAC ATC GCC ACC CCC CGT 3′	HEL-RdRp fragment amplification and sequencing of PCR product	metabion
Mar-Met1-F	5′ GCA GCT CTA AAT CCA TCC TTT GCA TCA TTC ATC TCC A 3′	MET fragment amplification and sequencing of PCR product	metabion
Mar-Met-R	5′ CCA CCC CAC AAG GCG GGA 3′	MET fragment amplification and sequencing of PCR product	metabion
Mar-Pro2-F	5' TCC CGC CTT GTG GGG TGG ATC CAT GCT 3'	PRO fragment amplification and sequencing of PCR product	metabion
Mar-Pro2-R	5' ACG GAA CGG GGG GTG GCG ATG TCA AGA 3'	PRO fragment amplification and sequencing of PCR product	metabion
MetPro-seq2-R	5′ AGG GTT CAG AAT CTG GGT TAG AAA TGG GAA C 3′	sequencing	metabion
MetPro-seq1-F	5′ TCC GCT CTC ACT TGG CTG CAA ATC ACA ACC AT 3′	sequencing	metabion
MetPro-seq1-R	5′ ATG GTT GTG ATT TGC AGC CAA GTG AGA GCG GA 3′	sequencing	metabion
Mar-CPL-F	5′TCT TAC TTC CAA CGA AAC TCC CGT 3′	Minor CP amplification	metabion
MarCP1F	5′ CCA ATG TCC AGA CCG GG CA 3′	Major CP amplification	metabion
MarCP2R	5′ TTA AGT GAA CTT TCC TCC AGA CGG T 3′	Minor and major CP amplification	metabion
MarBox1F	5′ GAG AGC GAA TTC TTT CCC TGG A 3′	"Marafibox" amplification	metabion
MarBox1R	5′ TGT GAG GTT TCT GGG GAG AGT CT 3′	"Marafibox" amplification	metabion
MARCP-short-Nde-F	5' TAC ATA TGG CCA ATG TCC AGA CCG GGC AGG A 3'	Major CP amplification for co-expression	metabion
MARCP-short-Xho-R	5' GAC TCG AGT TAA GTG AAC TTT CCT CCA GAC GGT 3'	Major CP amplification for co-expression	metabion
Mar-3UTR-RACE-R	5' GTA AGG TCT TGC TGG ACG ACC TTA TAC ACT 3'	CP-3′UTR fragment amplification and sequencing of PCR product	metabion
Mar-5UTR-RACE-F	5' GGT ATC TGT TTT TAC CCT CTT TCT AGC TTA ATC GCT 3'	5′UTR-PRO fragment amplification and sequencing of PCR product	metabion
SB-RdRp-CP-seq-F	5' TGG CGA GAT CCC GCT CTC TGT TCT CGA AGT CAT CTT 3'	RdRp-C-terminal part and CP fragment amplification	metabion

Table S2. The dataset used for evolutionary relationship analysis. Accessions of the selected publicly available *Tymoviridae* entries and the related sequence metadata are listed. Origin column: "E" indicates that the isolate was listed as an exemplar isolate for the species in ICTV virus metadata resource; "ICTV" indicates that the species is recognized in the official taxonomy of *Tymoviridae*, but no accession was present in virus metadata resource; "RefSeq" indicates that the virus does not yet have standing in the official virus taxonomy but was included in the analysis based on taxonomy associated with a RefSeq entry; "Nuccore>6k" indicates that the virus does not yet have a standing in the official virus taxonomy but was included in the analysis based on taxonomy associated with a complete or near-complete genome GenBank entry that was longer than 6000 bases in length.

(Please see separate excel file.)

Table S3. Descriptions of the generated MSA and features of the resulting trees.

(Please see separate excel file.)
